# Ultrasound Speckle Decorrelation Analysis‐Based Velocimetry for 3D‐Velocity‐Components Measurement Using a 1D Transducer Array

**DOI:** 10.1002/advs.202401173

**Published:** 2024-06-20

**Authors:** Yongchao Wang, Yetao He, Wenkai Chen, Jiyong Tan, Jianbo Tang

**Affiliations:** ^1^ Department of Biomedical Engineering Guangdong Provincial Key Laboratory of Advanced Biomaterials Southern University of Science and Technology ShenZhen Guangdong 518055 China

**Keywords:** 3D‐velocity‐components, blood flow imaging, dynamic analysis, ultrasound velocimetry

## Abstract

Ultrasound velocimetry has been widely used for blood flow imaging. However, the flow measurements are constrained to resolve the in‐plane 2D flow components when using a 1D transducer array. In this work, an ultrasound speckle decorrelation analysis‐based velocimetry (3C‐vUS) is proposed for 3D velocity components measurement using a 1D transducer array. The 3C‐vUS theory is first derived and validated with numerical simulations and phantom experiments. The in vivo testing results show that 3C‐vUS can accurately measure the blood flow 3D‐velocity‐components of the human carotid artery at arbitrary probe‐to‐vessel angles throughout the cardiac cycle. With such capability, the 3C‐vUS will alleviate the requirement of operators and promote disease screening for blood flow‐related disorders.

## Graphical abstract

The proposed ultrasound speckle decorrelation analysis‐based velocimetry (3C‐vUS) technique enables accurate measurement of the 3D velocity components and total flow speed of blood flow at arbitrary probe‐to‐vessel angles. This capability helps to alleviate the requirement of precise alignment and interpretation of operator, potentially mitigating the lack of skilled sonographers and facilitating large‐scale cardiovascular disease‐related screening at the community level.

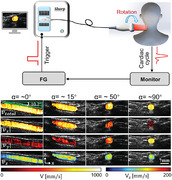

## 1 Introduction

Ultrasound imaging has become one of the most important diagnostic tools for a variety of clinical practices, such as prenatal screening,^[^ ^1^ ^]^ internal organ imaging,^[^ ^2^ ^]^ and circulation disease diagnosis.^[^ ^3^ ^]^ For the assessment of the circulation disease, the ultrasound‐based measurements of the blood vessel structure and blood flow dynamics provide a comprehensive evaluation of the blood flow circulation, making it a preferred tool for early disease screening.^[^ ^4^ ^]^ Particularly, measuring the blood flow velocity is not only useful to assess the regional flow status, but more importantly, it is also an information window reflecting the whole body's circulation condition. Hence, precise measurement of the absolute velocity is important for health state assessment, and it has drawn increasing attention in recent years.^[^ ^5^, ^6^, ^7^ ^]^

The conventional color Doppler imaging (CDI) can measure the axial velocity component by quantifying the frequency shift.^[^ ^8^ ^]^ However, the color Doppler‐based measurement is highly dependent on the imaging plane‐to‐vessel angle and the operator's alignment,^[^ ^8^ ^]^ making it challenging to accurately measure the flow speed. The vector Doppler imaging (VDI) is proposed to overcome the angle dependency of color Doppler by detecting the blood flow at two opposite angles.^[^ ^7^, ^9^, ^10^ ^]^ Despite its ability to measure the 2D in‐plane transverse and axial velocity components, the vector Doppler lacks the capability to resolve the through‐plane flow component, i.e., *v_y_*. In 1998, Jensen described a new method namely transverse oscillations (TOs),^[^ ^11^ ^]^ which measures the desired velocity component by introducing spatial oscillations along the corresponding direction and has been extensively investigated for the in‐plane flow velocity components measurement.^[^ ^12^, ^13^, ^14^ ^]^ Other techniques such as speckle tracking (ST) and particle imaging velocimetry are introduced to measure blood flow speeds but also limited to the in‐plane 2D velocity components measurement.^[^ ^15^, ^16^ ^]^ To resolve the 3D‐velocity‐components, intrinsic spectral broadening was proved to be associated with all 3D flow components and shows the potential to reconstruct the total flow speed.^[^ ^17^ ^]^ In 2015, Osmanski et al. proposed a Doppler spectral broadening‐based method to measure the through‐plane flow component.^[^ ^18^ ^]^ However, both the in‐plane and through‐plane transverse flow components (*v_x_* and *v_y_*) would result in frequency broadening, making it difficult to differentiate the specific contributions of the two transverse velocity components.

More recently, 2D transducer matrix‐based ultrasound imaging platforms have been used for 3D blood flow structure and velocity imaging, such as the 3D vector Doppler and 3D ultrasound localization microscopy (3D‐ULM).^[^ ^19^, ^20^, ^21^, ^22^ ^]^ However, it suffers from the challenges of low spatial resolution (3D vector Doppler) or extended data acquisition time as well as high demand for data processing (3D‐ULM). Besides, the 2D matrix probe requires multiple ultrasound platforms for simultaneous data acquisition, which greatly increases the cost and complexity.^[^ ^19^, ^21^ ^]^ Using fewer elements and channels with sparse matrix array or row‐column addressing transducers shows the potential to realize 3D flow imaging,^[^ ^23^, ^24^, ^25^, ^26^ ^]^ but it suffers from low signal‐to‐noise ratio and poor spatial resolution. Using a 1D transducer array to measure the 3D‐velocity‐components has the potential to address the aforementioned challenges. Zhou et al. introduced a multi‐technique‐based velocimetry to recover the 3D‐velocity‐components,^[^ ^27^, ^28^ ^]^ which combines the vector Doppler or ultrasound imaging velocimetry (UIV) to obtain the in‐plane speeds with the speckle decorrelation (SDC) analysis method for the measurement of the through‐plane speed. This method shows the potential to resolve the 3D‐velocity‐components using a 1D transducer array, but it requires two different data acquisition and processing protocols, making the measurement quite complicated.

In this work, we propose a novel ultrasound velocimetry method (3C‐vUS) to reconstruct the 3D‐velocity‐components (3C) *v_x,_ v_y,_* and *v_z_* using a 1D transducer array based on the ultrasound speckle decorrelation analysis. We rigorously derived the 3C‐vUS theory, which shows that the ultrasound signal decorrelation is determined by the 3D‐velocity components *v_x_, v_y_, v_z_*, and the imaging system's 3D point‐spread‐function (PSF). We further developed a comprehensive 3C‐vUS data processing algorithm, which first obtains the axial velocity component *v*_z_ from the phase change. Then, using the unique feature that the in‐plane lateral resolution (xPSF) can be modified by changing the beamforming apertures, two ultrasound speckle decorrelation functions were obtained from the same dataset. Thus, the transverse blood flow velocity components *v_x_* and *v_y_* can be differentiated and accurately reconstructed. The proposed 3C‐vUS technique was first validated with numerical simulation and phantom experiments. Then, we demonstrated its capability to accurately measure the blood flow 3D‐velocity‐components of human carotid artery at arbitrary probe‐to‐vessel angles throughout the cardiac cycle.

## 2 Result

### 2.1 Principle of the 3C‐vUS

**Figure 1:**
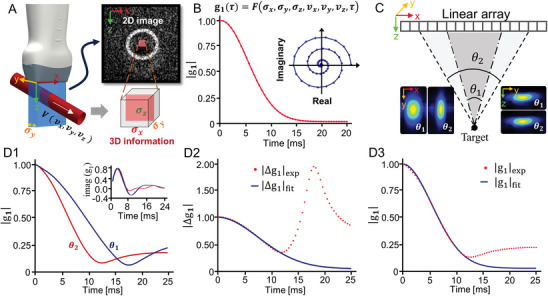
Principle of 3C‐vUS. A) The 2D image that is obtained with a 1D transducer array ultrasound probe contains 3D information. B) *g*_1_(τ) decorrelation in magnitude and in the complex plane (inset). C) Schematic diagram of using different receiving apertures during beamforming to obtain different in‐plane lateral resolutions (inset left). D) The data‐processing procedure for 3C‐vUS. D1) Magnitude decorrelation of *g*_1_(τ) for the same flow data but with different in‐plane lateral resolutions. D2) *v_x_* was recovered by fitting the ratio of the decorrelation functions Δ*g*_1_(τ). D3) *v_y_* was then reconstructed based on the obtained values *v_x_* and *v_z_* by fitting the decorrelation function of *g*_1, θ2_(τ).

As shown in **Figure** **1**A, the fundamental principle of reconstructing the 3D‐velocity‐components from a 2D ultrasound image obtained with a 1D transducer array is that the pixels in the 2D image are actually the projections of the 3D voxels along the through‐plane direction (Y). In other words, the ultrasound measurement voxel indeed contains 3D spatial and dynamic information, which were folded in the 2D pixels due to the lack of resolving ability in the Y direction. This fact lays the basis for the proposed 3C‐vUS technique to reconstruct the blood flow 3D‐velocity‐components using a 1D transducer array probe.

The principle of 3C‐vUS is based on the first‐order temporal autocorrelation function analysis of the ultrasound field signal, which is derived to be Equation 1. It is determined by the imaging system's point spread function (PSF) and the 3D‐velocity‐components of the blood flow.

(1) $$g_{1}\;\left(\tau \right)=\;e^{-\frac{{\left(v_{x}\tau \right)}^{2}}{4\sigma_{x}^{2}}-\frac{{\left(v_{y}\tau \right)}^{2}}{4\sigma_{y}^{2}}-\frac{{\left(v_{z}\tau \right)}^{2}}{4\sigma_{z}^{2}}}\cdot e^{i2k_{0}\tau v_{z}}$$

where *σ_x_*, *σ_y_* and *σ_z_* denote the Gaussian profile width at the 1/*e* value of the maximum intensity of the PSF in the x, y, and z directions, respectively; *v_x_*, *v_y_* and *v_z_* represent the 3D‐velocity‐components of the blood flow; and *k*_0_ is the wave number of the central frequency of the transducer. The magnitude of *g*_1_(τ) exhibits a semi‐exponential decay (Figure 1B), and a rotating decay in the complex plane (inset of Figure 1B). The fundamental features of *g*_1_(τ) function is that it decays faster for high‐speed flows, rotates at different paths for different flow angles, and more importantly, it's sensitive to both axial and transverse flows (independent of the Doppler angle), as shown in Figure S1 (Supporting Information). Therefore, the *g*_1_(τ) analysis has a great potential for flow measurement. For more details regarding the theoretical derivation of Equation 1, please refer to the Supporting Information.

One may note from Equation 1 that the phase change of *g*_1_(τ) is determined by the axial velocity component *v_z_*, while the amplitude decorrelation of *g*_1_(τ) is affected by all 3D‐velocity‐components. Therefore, the axial velocity component *v_z_*, can be reconstructed from the phase information, but there exists a crosstalk when reconstructing *v_x_* and *v_y_* using Equation 1 as these two terms both contribute to the magnitude decorrelation of *g*_1_(τ). To address this issue, we propose to utilize the unique feature in ultrasound imaging that the in‐plane lateral resolution (xPSF) can be changed by using different beamforming apertures (Figure 1C). Thus, the *g*_1_(τ) functions with different decorrelation rates can be obtained from the same dataset by changing the beamforming apertures (Figure 1D1).

(2)

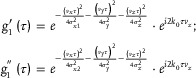

where *σ_x1_* and *σ_x2_* denote the Gaussian profile width at the 1/e value of the maximum intensity of the modulated xPSF. By dividing $g_{1}^{\prime }\left(\tau \right)$ with 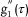 in Equation 2, we can obtain a Δ*g*_1_(τ) term, which is determined by only one unknown variable *v_x_* and the known values *σ_x1_* and *σ_x2_*.

(3) $$\Delta g_{1}\;\left(\tau \right)=e^{-\frac{{\left(v_{x}\tau \right)}^{2}}{4\sigma_{x1}^{2}}+\frac{{\left(v_{x}\tau \right)}^{2}}{4\sigma_{x2}^{2}}}\;$$

Hence, as depicted in Figure 1D2, a least square nonlinear fitting procedure can be applied to fit the Δ*g*_1_(τ) to reconstruct *v_x_*. With the obtained in‐plane transverse and axial velocities (i.e., *v_x_* and *v_z_*), we can further resolve the through‐plane velocity *v_y_* by applying a nonlinear fitting procedure on the original $g_{1}^{\prime }\left(\tau \right)$ (Figure 1D3). Finally, the total velocity of blood flow could be obtained with Equation 4. The data processing algorithm is summarized in Figure S2 (Supporting Information).

(4) $$v_{\mathrm{total}}=\sqrt{v_{x}^{2}+v_{y}^{2}+v_{z}^{2}}\;$$

### 2.2 The Influence of Beam Forming Aperture on System's PSF and ***g***_1_(***τ***) Decorrelation

We first performed a static phantom experiment to characterize the effect of changing the receiving aperture during ultrasound image beamforming on the system's PSF and *g*_1_(τ) decorrelation. First, crystal balls with a diameter of ≈50 µm were fixed in a phantom sample and scanned along the Y (through‐plane) direction. The detected echo signal was beamformed into B‐mode images, and finally these B‐mode images were applied to reconstruct the 3D images of crystal balls, which represents the system's 3D PSF as the size of the crystal balls is much smaller than the system's 3D PSF ([0.5, 1.0, 0.4] mm).

**Figure 2:**
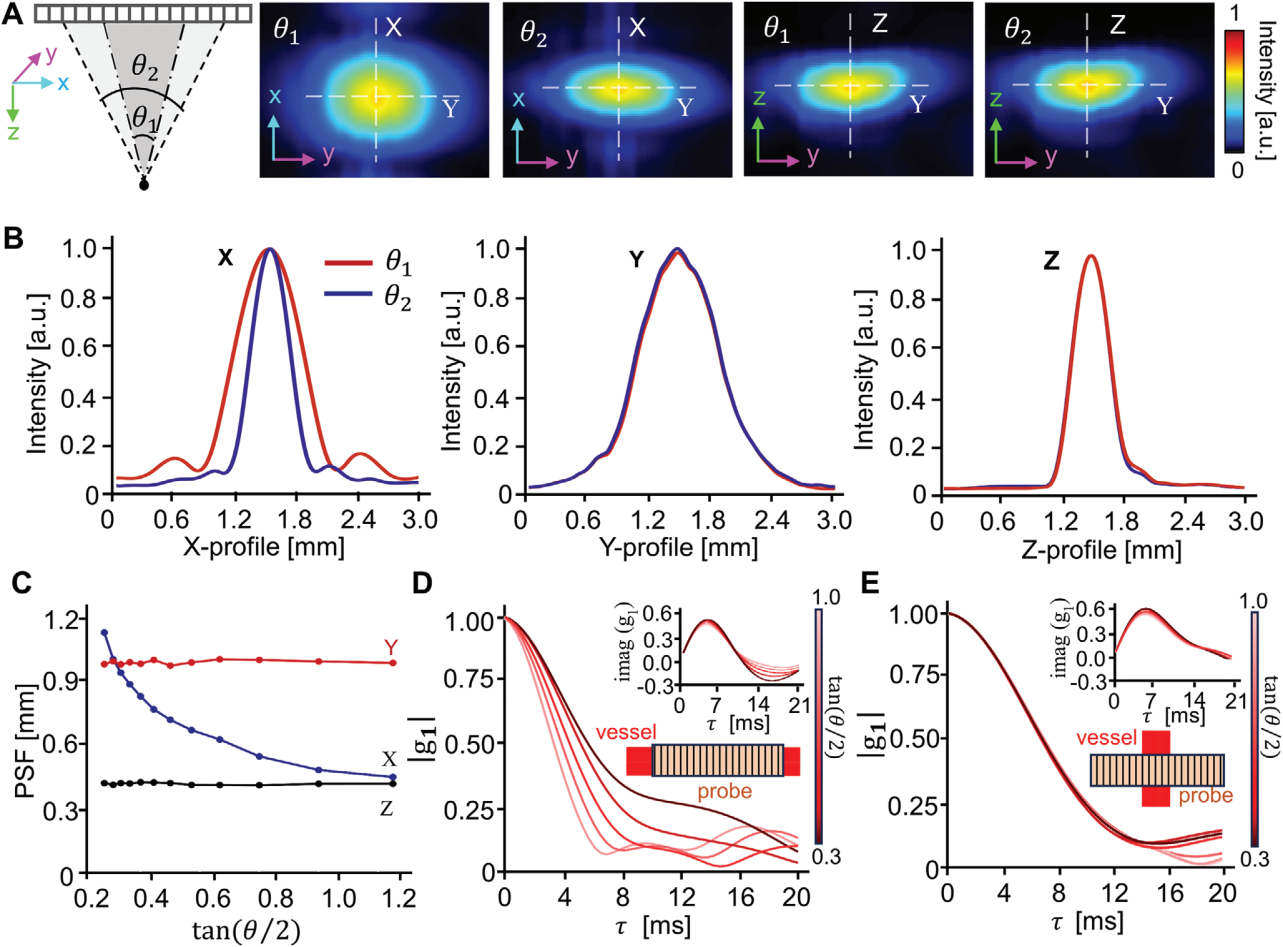
Influence of beamforming aperture on PSF and *g*_1_(τ) function. A) Images of crystal ball in X–Y and Y–Z planes reconstructed with different receiving apertures. B) Profiles (along X, Y, Z directions, respectively) extracted from central line marked with dotted white lines in A. C) The 3D point‐spread‐function (PSF) obtained at different beamforming apertures. D) *g*_1_(τ) obtained with different beamforming apertures (i.e., different xPSF). The vessel is aligned with the probe detection plane. E) *g*_1_(τ) obtained with different beamforming apertures (i.e., different xPSF). The vessel is aligned perpendicular to the probe detection plane.

**Figure** **2**A,B shows details regarding the calibration of the system's 3D PSF for different beamforming apertures (𝜃). From Figure 2B, we see that increasing the receiving aperture does not affect the PSF in Z and Y directions but improves the resolution in X direction. Quantitatively, the in‐plane lateral resolution gradually improves from 1142 to 472 µm with the increasing receiving aperture, while the elevational and axial resolution remain at ≈1028 and ≈420 µm, respectively, as shown in Figure 2C. This suggests that the receiving aperture only changes the in‐plane lateral resolution (xPSF), making it possible to obtain different *g*_1_(τ) functions from the same dataset by changing the beamforming aperture.

Further, we investigated the influence of receiving aperture on *g*_1_(τ) decorrelation by calculating *g*_1_(τ) function from the same flow signal but beamformed with different apertures. Figure 2D shows the scenario where the probe's imaging plane is aligned with the vessel. We see that the magnitude of *g*_1_(τ) decays faster when applying a larger receiving aperture, i.e., a smaller xPSF, while all the *g*_1_(τ) have a similar period (phase) in the imaginary plots (inset), suggesting that it does not affect the axial speed measurement (phase) when changing the receiving aperture. Figure 2E further shows the scenario where the probe is perpendicular to the vessel, in which case, the modulation of the in‐plane lateral resolution (xPSF) does not affect the *g*_1_(τ) decorrelation. Those facts suggest that changing the in‐plane lateral resolution (xPSF) would only affect the decorrelation contributed by the in‐plane lateral flow velocity component.

### 2.3 Numerical Simulation

**Figure 3:**
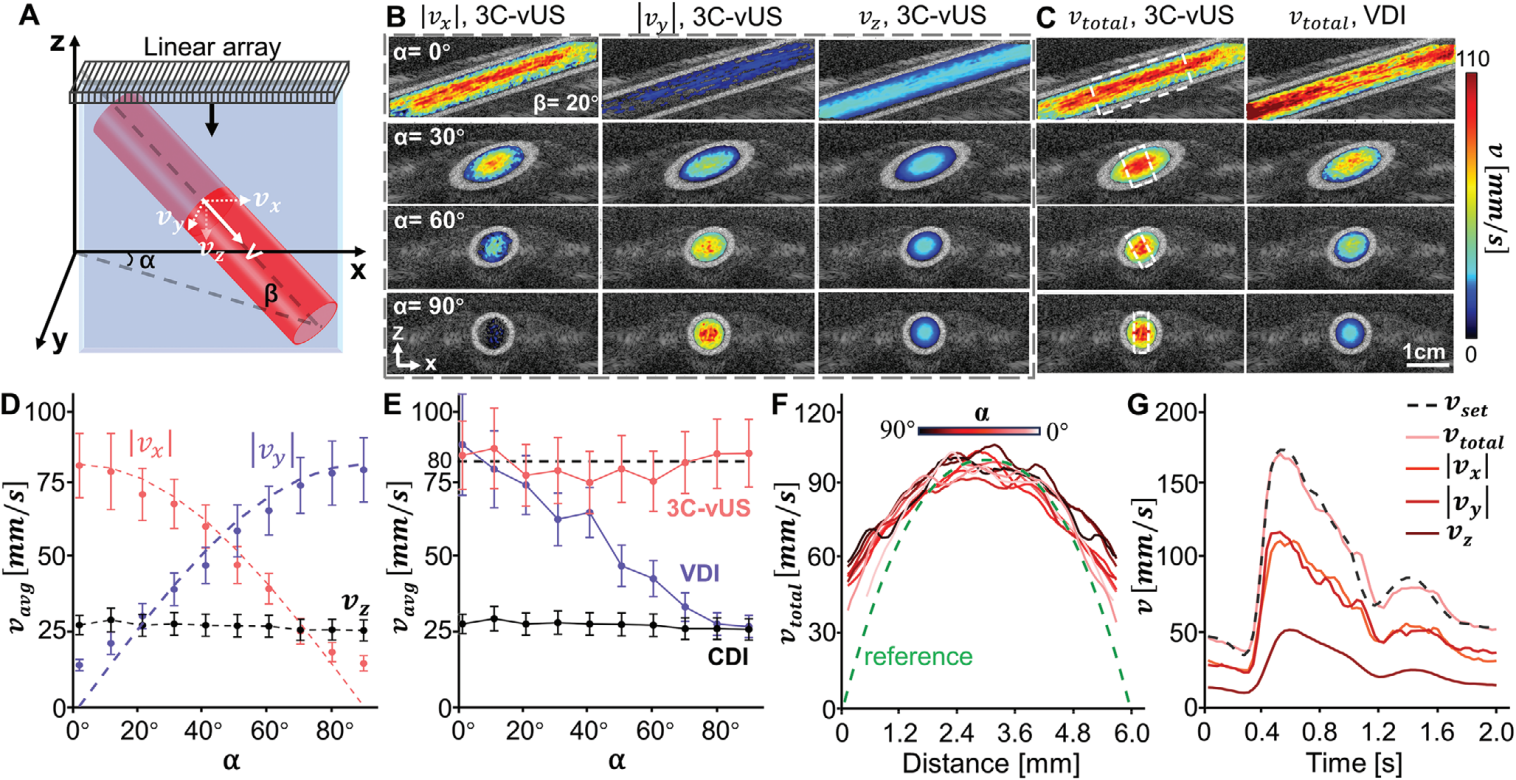
Numerical validation. A) A blood vessel tube phantom was imaged with a 1D transducer probe at a probe‐to‐vessel angle of *α*. (*β* = 20°). B) Representative *v_x_*, *v_y_*, and *v_z_* maps obtained with 3C‐vUS at 0°, 30°, 60° and 90°, respectively. C) Total blood flow velocity obtained with 3C‐vUS (left column) and results obtained with vector Doppler (right column). D) The averaged *v_x_*, *v_y_*, and *v_z_* measured at 10 probe‐to‐vessel angles. E) Comparison of cross‐sectional averaged total speed measured by 3C‐vUS, color Doppler imaging (CDI) and vector Doppler imaging (VDI) for different angle *α*. F) Total velocity profiles extracted from positions marked with gray dotted squares in (C). G) The total speeds, and 3D‐velocity‐components obtained with 3C‐vUS for a simulated pulsation flow.

For numerical validation, we first tested the performance of 3C‐vUS to differentiate probe‐to‐vessel angles (α, ranging from 0° to 90°), in which a tilted flow (tilted angle: *β* = 20°) which has a preset Gaussian velocity profile with a peak speed of 100 mm s^−1^ was simulated with Field II simulation toolkit. Note that the probe‐to‐vessel angle refers to the angle between the projections of the ultrasound probe and the blood vessel on the x‐y‐plane, as shown in **Figure** **3**A. Figure 3B shows the 3D‐velocity‐components maps obtained at the probe‐to‐vessel angle of *α* = 0°, 30°, 60° and 90°, respectively. Qualitatively, we see that the obtained *v_x_* gradually decreases while *v_y_* gradually increases with angle α, and the measured axial velocity component *v_z_* is ≈26 mm s^−1^ for all cases. Figure 3D shows the average results with standard deviation of the 3D velocity components at 10 probe‐to‐vessel angles (*α*). We notice that the *v_x_* and *v_y_* follow the cosine and sine distribution with angle α, respectively, which agrees well with the expectation (dashed curves in Figure 3D), suggesting that 3C‐vUS can differentiate *v_x_* and *v_y_* at different probe‐to‐vessel angles. We also validated the accuracy for speed measurements by changing the preset flow speed from 25 mm s^−1^ up to 600 mm s^−1^, as shown in Figure S3 (Supporting Information).

As the acquired raw dataset can also be used to calculate the color Doppler and vector Doppler, we compared the measurements that were obtained with these three methods (Figure 3C,E). As shown in Figure 3E, we notice that the CDI‐based measurement strongly depends on the tilted angle (*β* = 20°), which has a measured value of ≈26 mm s^−1^ for all probe‐to‐vessel angles. The VDI can overcome the angle‐dependency of color Doppler for the well‐aligned in‐plane detection, but the measured total speeds (*v*_total_) gradually deviate from the real value when the probe‐to‐vessel angle α changes (Figure 3E). In contrast, the average *v*_total_ obtained with 3C‐vUS is around 80 mm s^−1^ for all cases with an average error of −4.32 ± 3.53%. In addition, the laminar flow profile is observed, as shown in Figure 3F, where all the measured total speeds have a similar flow profile with a peak value ≈100 mm s^−1^, which agrees well with the preset flow (green dashed curve in Figure 3F). The non‐zero value obtained near the vessel wall is due to the limited spatial resolution and the employed clutter rejection method, as we discussed in Figure S4 (Supporting Information). We also applied 3C‐vUS to measure the blood flow velocities with pulsatile flow, as shown in Figure 3G and Movie S1 (Supporting Information). It is observed that the measured speeds (*v*_x_, *v*_y_, *v*_z_ and *v*_total_) have a similar temporal pulsatile pattern to the preset flow (dashed gray curve). These results suggest that the 3C‐vUS can accurately obtain the flow's total speed at arbitrary probe‐to‐vessel angle.

Further, we performed a series of tests to show the effects of frame rate (Figure S5, Supporting Information), period window size (data acquisition window size, Figure S6, Supporting Information), the fitting window size (Figure S7, Supporting Information), 3D PSF (Figure S8, Supporting Information) and noise (Figure S9, Supporting Information) on the velocity measurement using 3C‐vUS. Our conclusion is that a higher frame rate is preferred for a more accurate fitting of the decorrelation function, the 3C‐vUS is resistive to noise, the data acquisition window size shall be at least 400 repeats (26 ms), and the fitting window size shall be longer than the decorrelation time (a 60‐repeats, 4 ms was used in this study). Please refer to the Supporting Information.

### 2.4 Phantom Validation

**Figure 4:**
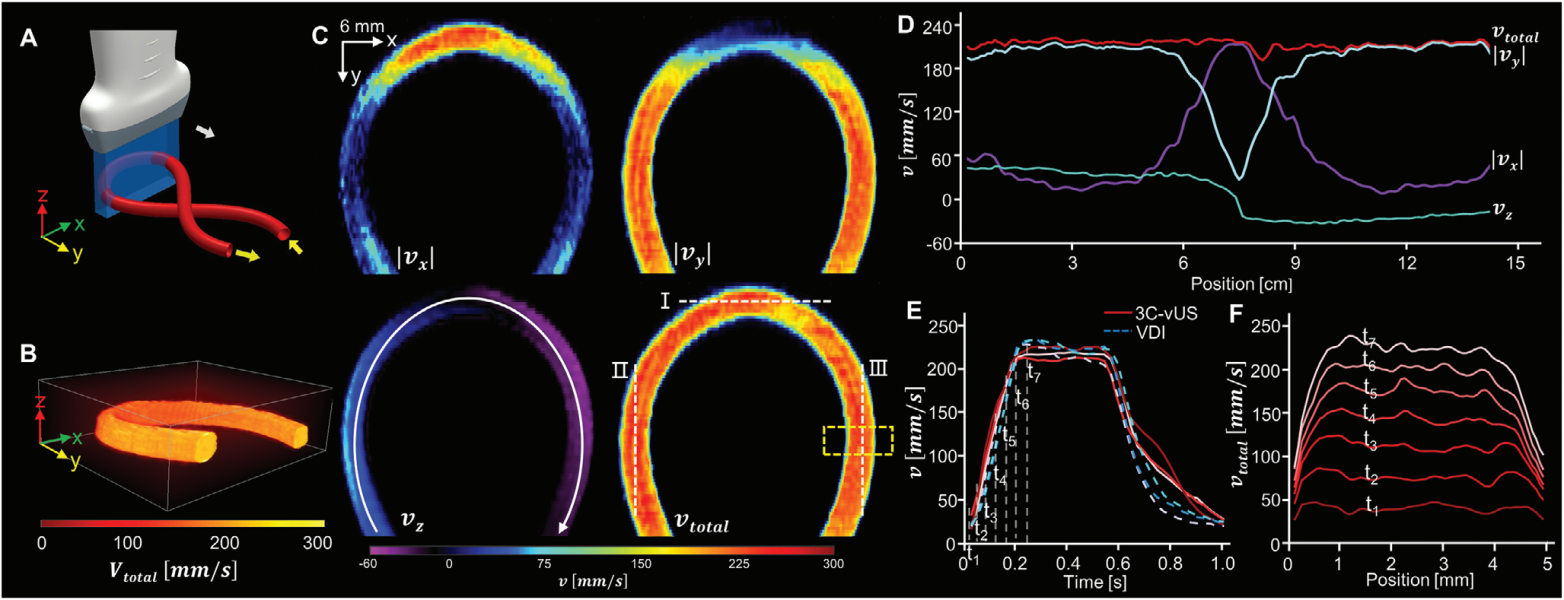
Phantom validation. A) A homemade circular‐shaped flow phantom was imaged with a 1D transducer probe at different transverse locations (Y). B) The 3D representation of the obtained total velocity. C) The mean value projections in the XY plane of *v_x_*, *v_y_*, *v_z_*,and total velocity (*v*_total_), respectively. D) The obtained *v_x_*, *v_y_*, *v_z_* and *v*_total_ values along the trajectory of the midline of the curved tube, indicated with white arrow in the *v_z_* map of Figure 4C. E) The time‐varying *v*_total_ curves measured with vector Doppler and 3C‐vUS at locations marked with white dashed lines in the *v*_total_ map of Figure 4C. F) The *v*_total_ cross‐sectional profile measured at different time‐points indicated in Figure 4E, which corresponds to different preset speeds.

To validate the capability of 3C‐vUS, we designed a phantom sample which has a circular‐shaped plastic tube (diameter 5 mm) buried in a static background. A solution made of 1% Silicon dioxide (SiO_2_) suspension was pumped through the plastic tube. A 1D linear ultrasound probe (center frequency 7.2 MHz) was carried by a linear stage to measure the 3D‐velocity‐components at different cross sections, as shown in **Figure** **4A**. Figure 4B and Movie S2 (Supporting Information) show the volumetric 3D representation of the measured total speed.

The en face view of the corresponding mean value projections of the 3D‐velocity‐components and the total velocity are shown in Figure 4C. Figure 4D presents the corresponding measured values (*v*_total_, *v_x_*, *v_y_*, and *v_z_*) along the middle line of the plastic tube (white dashed curve in the *v_z_* map of Figure 4C). We see that at the top of the circular‐shaped plastic tube, where the probe‐to‐vessel angle α is close to 0°, the measured *v_x_* is about 220 mm s^−1^; while the corresponding *v_y_* reaches the minimum. When moving the 1D linear ultrasound probe from the top to the middle point of the plastic tube phantom, the probe‐to‐vessel angle α gradually increases to 90°, and the measured *v_y_* increases to 225 mm s^−1^ and *v_x_* reaches the minimum, respectively (Figure 4D). One may also notice that the measured *v_z_* gradually changes along the middle line of the flow and the axial flow direction reverses at the top region of the tube. This is due to the alignment of the plastic tube with outflow higher than the inflow which leads to a gradually changing beam‐to‐flow angle, as shown in Figure 4A. In addition, the measured total speed (red, Figure 4D) along the middle line of the flow is very constant for different positions (i.e., different probe‐to‐vessel angles). Therefore, at arbitrary probe‐to‐vessel angles, the 3C‐vUS can not only accurately measure the speeds of (*v_x_*, *v_y_*, *v_z_*, and *v*_total_) but also determine the axial flow direction.

In addition, we further conducted an experiment with pulsatile flow to verify the capability of 3C‐vUS for the measurement of time varying velocities. We used the vector Doppler velocimetry as the comparison standard and since it can only accurately recover the well aligned in‐plane speed, we acquired vector Doppler data at the three locations indicated by the white dashed lines in the *v*_total_ map of Figure 4C. Figure 4E shows the obtained temporal *v*_total_ speeds measured with 3C‐vUS and the vector Doppler method (VDI). We notice that the total speeds measured at different locations have similar value throughout the pulsatile cycle and agree well with the total speeds obtained with VDI (Figure S10, Supporting Information), suggesting that the 3C‐vUS can accurately measure the velocity of a changing flow. Figure 4F shows the flow profile at different time points marked in Figure 4E (corresponding to different preset speeds). Laminar flow profiles are observed, especially for higher‐speed flows.

### 2.5 In Vivo Measurement of Human's Carotid Artery

**Figure 5:**
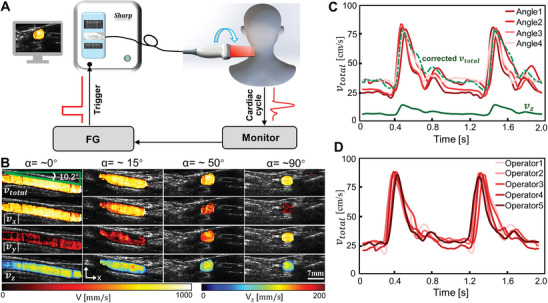
In vivo imaging of human carotid artery. A) Experimental setup for carotid artery imaging. B) The measured blood flow 3D velocity maps of the carotid artery during systolic state at five different probe‐to‐vessel angles. C) Time courses of total blood flow velocity obtained within two cardiac cycles for the different probe‐to‐vessel angles. D) Time courses of total blood flow velocity for the same subject but acquired by five untrained operators.

We further applied 3C‐vUS to measure the blood flow velocities of the common carotid artery from a health subject. Since only the blood flow area is the interested region, we used the calculated *g*_1_(τ) function to make a spatial mask for the blood flow region to reduce the data processing time, as discussed in the Experimental Section and Figure S11 (Supporting Information). **Figure** **5**A presents the experimental diagram for data acquisition, in which the 1D transducer array rotated along the arrow direction to measure the blood flow speed of the carotid artery at different probe‐to‐vessel angles. Figure 5B presents the speed maps of the total velocity and the 3D‐velocity‐components obtained at the systolic period for different probe‐to‐vessel angles. The obtained *v*_total_ at the four angles during the systolic period were 869 ± 63, 845 ± 75, 858 ± 73, and 863 ± 67 mm s^−1^, respectively. The measured *v*_total_ agrees with the reference value of ≈900 mm s^−1^ for systolic state (Volunteer with diameter stenosis less than 20%) and the color Doppler recovered *v*_total_ (𝛼 = 0°, 𝛽 = 10.2°).^[^ ^29^, ^30^, ^31^ ^]^ Similarly, as presented in Figure S12 (Supporting Information), the *v*_total_ measured at diastolic state were 246 ± 53, 258 ± 75, 297 ± 56, and 314 ± 51 mm s^−1^, respectively, agreeing well with the reference value of ≈270 mm s^−1^ and the color Doppler recovered *v*_total_ (𝛼 = 0°, 𝛽 = 10.2°).^[^ ^29^, ^30^, ^31^ ^]^ Figure 5C and Movies S3–S6 (Supporting Information) present the time traces of the *v*_total_ obtained at four different probe‐to‐vessel angles. We see that the *v*_total_ changes periodically, demonstrating the capacity of 3C‐vUS to precisely measure the total speed of carotid blood flow throughout the cardiac cycle and at arbitrary probe‐to‐vessel angles.

In addition, to show that the 3C‐vUS is independent of operators, five untrained students were guided to acquire data from the same volunteer. The *v*_total_ obtained with the five untrained operators is shown in Figure 5D. We can see that the *v*_total_ acquired by different operators has similar result within two cardiac cycles, and the corresponding systolic and diastolic *v*_total_ (850 mm s^−1^ and 250 mm s^−1^, respectively) were comparable with reference value.^[^ ^29^, ^30^, ^31^ ^]^ This test suggests that the 3C‐vUS is not dependent on the operator and his/her experience, which can greatly alleviate the requirement for operators, hence promoting wide adoption of this technique for pre‐clinical screening.

## 3 Discussion

In this work, we introduced a novel ultrasound speckle decorrelation analysis technique to reconstruct the 3D blood flow velocity components from the 2D ultrasound image that was obtained with a 1D transducer array. A major innovation of the proposed technique is that it enables to differentiate the transverse velocity components (i.e., *v_x_* and *v_y_*) by utilizing the unique feature in ultrasound image beamforming that the in‐plane lateral resolution (xPSF) can be modified by applying different beamforming apertures. As reported in previous study, although SDC based velocimetry has shown its potential for axial velocity measurement, it still suffers from the crosstalk when reconstructing the transverse velocity components.^[^ ^32^ ^]^ By changing the in‐plane lateral resolution, we obtained two first order field autocorrelation functions ($g_{1}^{\prime }\left(\tau \right)$ and 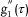) from the same data set, which helps to differentiate the decorrelation contribution of the in‐plane lateral speed *v_x_* from the through‐plane speed *v_y_*. With a two‐step nonlinear fitting procedure and phase analysis, all 3D‐velocity‐components (*v_x_*, *v_y_* and *v_z_*) of the flow can be further accurately reconstructed.

We validated our method with numerical simulation and phantom experiments. The results suggest that this method allows accurate measurement of the 3D‐velocity‐components at different probe‐to‐vessel angles. Compared to vector Doppler, the total speed measured with 3C‐vUS agrees well with preset speeds (average error: −4.32 ± 3.53%), and the velocity components in the lateral X and Y directions were consistent to the probe‐to‐vessel angles. Further, we applied this method for 3D blood flow velocity measurement of human's common carotid artery using a regular 7.2 MHz center frequency 1D transducer probe. The results showed that the time traces of total velocity measured at four probe‐to‐vessel angles have similar measurement throughout the cardiac cycles, and the total speeds measured at the systolic and diastolic states agree well with the known values and the color Doppler recovered *v*_total_ (*α* = 0°), suggesting that the 3C‐vUS can accurately measure the blood flow velocity from human subjects. Finally, we tested how robust is 3C‐vUS for speed measurement by guiding untrained students to acquire data from a health volunteer. The obtained results showed that similar total speeds were acquired by different students, which suggests that the proposed 3C‐vUS technique is independent of operator and his/her experience.

For 3C‐vUS, the in‐plane velocity components (i.e., *v_z_*, *v_x_*) are first obtained and used as prior knowledge for through‐plane component (i.e., *v_y_*) measurement, leading to the reliability of *v_y_* is contingent upon the determination of the pre‐obtained *v_z_* and *v_x_*. Thus, to test whether the in‐plane components obtained with 3C‐vUS is accurate, a detailed comparison was conducted among the 3C‐vUS, Transverse oscillations (TOs), vector Doppler (VDI) and Speckle tracking (ST) techniques using the open‐source MUST toolbox.^[^ ^33^, ^34^, ^35^ ^]^ As shown in Figures S13 and S14 (Supporting Information), the in‐plane vector velocity maps and the quantitative results demonstrate that 3C‐vUS provides comparable in‐plane velocity measurement (deviation less than 6.49%) to traditional TOs, VDI and ST for all beam‐to‐angle cases, suggesting that 3C‐vUS is accurate in measuring the in‐plane blood flow velocity components.

Note that despite the ultrasound intensity‐based speckle decorrelation analysis (SDC) can be combined with VDI and ST to obtain the 3D‐velocity‐components, the advantage of the proposed 3C‐vUS is that it enables the measurement of all 3D‐velocity‐components with a single data acquisition and processing strategy (*g*_1_(τ) analysis model) when using a 1D linear transducer probe. No other techniques (i.e., VDI, UIV) are needed for the in‐plane components analysis, which helps to simplify the experimental protocol and data processing. In addition, the 3C‐vUS supports a higher data acquisition rate as it does not require multi‐angle plane wave emission, which theoretically increases its maximum measurable speed (see details in below discussion). More importantly, the 3C‐vUS can be applied for any 1D linear transducer probe as long as its 3D PSF can be correctly calibrated.

It's worth noting that a high data acquisition rate is often preferred when using the 3C‐vUS technique. As discussed in Figure S5 (Supporting Information), insufficient frame rate would result in fewer sampling points in the decorrelation period for the non‐linear fitting, which would affect the estimation accuracy of the total flow speed, especially for high‐speed flows. With ultrafast plane wave imaging,^[^ ^36^ ^]^ the maximum frame rate is now limited by the flight time of the ultrasound wave for expected imaging depth, which is up to ≈30 kHz for an image depth of 25 mm. Note that excessively increasing the frame rate to improve the temporal resolution is not recommended due to the potential heating damage to the probe. Another way to increase the sampling points of *g*_1_(τ) within the decorrelation period is to enlarge the system's 3D PSF so that more time is needed for a particle to flow through the resolution voxel (Figure S8 Supporting Information). In this work, to measure the carotid artery speed in the systolic state which is almost 1 m s^−1^, we applied a 7.2 MHz 1D ultrasound probe with a modulated 3D PSF of (0.5, 1.0, 0.4) mm. Indeed, an ultrasound probe with a lower 3D PSF can be used to measure the fast flow speed in large blood vessels.

Although the 3C‐vUS exhibits a great potential in flow speed measurement, there exists two main limitations. As previously reported,^[^ ^37^, ^38^ ^]^ visualizing the complex blood flow patterns, particularly the 3D velocity vector fields, has shown to provide valuable information for the assessment of circulation conditions. For example, the analysis of 2D/3D flow patterns in the left ventricle has provided an insight of cardiac functions and is extensively utilized in diagnosing heart diseases, including valve stenosis and regurgitation. Moreover, disrupted flow patterns (i.e., reverse flow, turbulent flow) were proven to be associated with the presence of atherosclerotic plaque, providing extra measures for the diagnosis of carotid stenosis. However, the 3C‐vUS can only determine the axial flow direction (downward or upward) based on the rotation direction (clockwise or anticlockwise) of *g*_1_(τ) in the complex plane. As observed from Equation 1, the transverse velocity components (i.e., *v_x_*, *v_y_*) are squared in *g*_1_(τ) model, which means that the flow direction in transverse plane would not affect the *g*_1_(τ) decorrelation, making it difficult to differentiate the transverse flow direction. Despite it has no influence on the measurement accuracy of total blood flow speed, it may hinder the ability to identify disrupted flow patterns associated diseases. Thus, additional improvement shall be taken into account to determine the transverse flow direction. One potential strategy to determine the transverse flow direction using 3C‐vUS is to combine the axial velocity direction with the geometry of the blood vessel, as illustrated in Figure S15 (Supporting Information). Another limitation is that the 3C‐vUS is not suitable for the measurement of microvascular network, such as the brain where exists dense small vessels and the diameters of those vessels are typically less than 100 µm. The speed estimation would be incorrect when enlarging the in‐plane PSF as more vessels may be included in the larger detection voxel compared to the original resolution. Thus, the 3C‐vUS is suitable for the measurement of 3D velocity components of the large vessels.

It has been demonstrated that the increased blood flow velocity in major arteries is an important biomarker of the vessel stenosis, and it has been used for the prediction of the risk of arteriosclerosis‐induced stroke.^[^ ^30^, ^31^ ^]^ The 3C‐vUS, which enables accurate measurement of total flow speed, can offer valuable reference to determine the degree of artery stenosis, guiding the clinician in precise diagnostic and optimal treatment decisions. Moreover, by alleviating the requirement of well‐trained clinicians, 3C‐vUS simplifies the data‐acquisition protocol of blood flow speed measurement, potentially mitigating the lack of skilled sonographers and facilitating large‐scale cardiovascular disease‐related screening at the community level.

To summarize, the proposed 3C‐vUS technique can accurately measure the 3D blood flow velocity components by using a traditional 1D ultrasound transducer array. The speed components can be measured at arbitrary probe‐to‐vessel angles. This ability is significant for clinical practices as it doesn't require well‐trained operators, which holds the potential for wide adoptions in circulation‐related early disease screening.

## 4 Experimental Section

### Experimental Setup and Data Acquisition

A commercial ultrafast ultrasound platform (Prodigy 256, S‐sharp, Taiwan, China) with a 128‐element linear transducer probe (L11‐5v, JIARUI, Shenzhen, China) was used for plane wave emission and detection. The Prodigy 256 platform supports an ultrafast pulse rate frequency (PRF) up to 30 kHz. The linear array has a center frequency of 7.2 MHz with a bandwidth of 92%.

### 3C‐vUS Data Processing

Figure S2A (Supporting Information) summarizes the 3C‐vUS data processing procedure. Based on the developed 3C‐vUS theory, B‐mode images with different lateral PSFs were reconstructed with two receiving apertures (Figure S2B,C, Supporting Information) and were further used to obtain 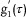 and 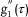 (Figure S2D, Supporting Information). Then, the 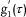 is divided by 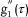 to obtain a new term Δ*g*_1_(τ).

(5)

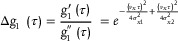




Note that Δ*g*_1_(τ) is determined by the unknown variable *v_x_* and the known values *σ_x1_* and *σ_x2_*. Therefore, a least square nonlinear fitting procedure was employed to fit Δ*g*_1_(τ) to obtain the *v_x_*. Specifically, a series of *v_x_* values were tested to find the *v_x_* that maximizes the coefficient of determination *ΔR*, which is defined in Equation 6 and acts as the objective function for a constrained least squares regression nonlinear fitting procedure to estimate *v_x_*.

(6) $$\Delta R\;=\;1-\frac{\left\{{\left|\Delta g_{1,\exp}\left(\tau \right)-e^{-\frac{{\left(v_{x}\tau \right)}^{2}}{4\sigma_{x1}^{2}}+\frac{{\left(v_{x}\tau \right)}^{2}}{4\sigma_{x2}^{2}}}\right|}^{2}\right\}}{{\left\{\left|\Delta g_{1,\exp}\left(\tau \right)-\left\{\Delta g_{1,\exp}\left(\tau \right)\right\}\right|\right\}}^{2}}$$

where Δ*g*_1,exp_(τ) is the experimental decorrelation function calculated with Equation 5; {…} denotes the temporal ensemble averaging; |…| denotes the absolute value. Note that the |*g*_1_(τ)| signal is noisy and useless after the decorrelation period (Figure 1D1 and Figure S2D, Supporting Information). Hence, a proper fitting period should be determined. In this work, the time lag was used when 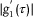 (higher lateral resolution) reaches its first minimum as the fitting period for data processing.

The axial velocity *v_z_* could be resolved from the phase change of 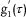, as the phase change is determined by the *v_z_* according to Equation 1. With the calculated in‐plane velocity components (*v_x_* and *v_z_*), the through‐plane velocity (*v_y_*) was further resolved by applying a nonlinear fitting procedure on the original $g_{1}^{\prime }\left(\tau \right)$. Specifically, the optimal *v_y_* was determined by finding the value that maximizes the coefficient of reference factor *R*, which is defined in Equation 7 and acts as the objective function for a constrained least square regression nonlinear fitting procedure to estimate *v_y_*.

(7) $$R\;=\;1-\frac{\left\{{\left|g_{1,\exp}^{\prime }\left(\tau \right)-e^{-\frac{{\left(v_{x}\tau \right)}^{2}}{4\sigma_{x}^{2}}-\frac{{\left(v_{y}\tau \right)}^{2}}{4\sigma_{y}^{2}}-\frac{{\left(v_{z}\tau \right)}^{2}}{4\sigma_{z}^{2}}}\cdot e^{i2k_{0}\tau v_{z}}\right|}^{2}\right\}}{{\left\{\left|g_{1,\exp}^{\prime }\left(\tau \right)-\left\{g_{1,\exp}^{\prime }\left(\tau \right)\right\}\right|\right\}}^{2}}$$

where $g_{1,\exp}^{\prime }\left(\tau \right)$ was calculated with experimental data.

### Adaptive Singular Value Decomposition Filtering

To remove the dominant background signal and extract blood blow dynamic information, the singular value decomposition (SVD)‐based spatiotemporal filtering method was applied for clutter rejection.^[^ ^39^ ^]^ The lower rank (*N*_thres_) of SVD filtering was determined by finding the first highly correlated square in the spatial similarity matrix.^[^ ^40^ ^]^ The largest *N*_thres_ singular value components that mainly represented background tissue signal were removed:

(8) $$\mathrm{sIQ}\;\left(x,z,t\right)=\;\sum_{N_{\mathrm{thres}}}^{N_{r}}\lambda_{i}U_{i}\left(x,z\right)V_{i}\left(t\right)$$

where sIQ*(x, z, t)* is the clutter rejected ultrasound signal; *U_i_* and *V_i_* are the *i* ^th^ columns of the spatial singular matrix and temporal singular matrix, respectively; *λ* is the descending‐ordered singular values; *N_r_* is the upper rank of the raw dataset.

### Color Doppler Data Processing

After bulk motion rejection using the adaptive SVD filtering, the Doppler velocity was obtained by calculating the Doppler frequency shift.^[^ ^5^ ^]^

(9) $$v_{\mathrm{cz}}=\;-\frac{v_{\mathrm{sound}}}{2f_{0}}\frac{\int_{-f_{s}/2}^{f_{s}/2}f{\left|F\left(\mathrm{sIQ}\right)\right|}^{2}df}{\int_{-f_{s}/2}^{f_{s}/2}{\left|F\left(\mathrm{sIQ}\right)\right|}^{2}df}$$

where *v*_cz_ is the Doppler velocity; *v*_sound_ is the speed of sound in the medium; *f*_0_ denotes the center frequency of the linear array; sIQ is the clutter rejected complex ultrasound signal; *f*_s_ is the frame rate which equals to the sampling frequency of sIQ; *F* represents the Fourier transform.

### Vector Doppler Data Processing

The Doppler velocity measured at each emission angle could be decomposed into one component, *v*_x_ along the lateral direction and the other component, *v*_z_ along the axial direction through the triangular transformation, which is described as:

(10) $$v_{z}\cdot \cos\left(\theta_{i}\right)+v_{x}\cdot \sin\left(\theta_{i}\right)\;=V_{\mathrm{cz},\theta_{i}}\;$$

where *θ_i_* is the *i*th emission angle; $V_{\mathrm{cz},\theta_{i}}$ is the Doppler velocity of *i*th emission angle. A least‐square fitting procedure was employed to determine the in‐plane velocities (*v_x_* and *v_z_*) from N angles measurement by:^[^ ^41^ ^]^

(11) $$\left[\begin{array}{cc} \cos\left(\theta_{1}\right)+1 & \sin\left(\theta_{1}\right)\\ \begin{array}{c} \cos\left(\theta_{2}\right)+1\\ \vdots \end{array} & \begin{array}{c} \sin\left(\theta_{2}\right)\\ \vdots \end{array}\\ \cos\left(\theta_{N}\right)+1 & \sin\left(\theta_{N}\right) \end{array}\right]\;\left[\begin{array}{c} v_{z}\\ v_{x} \end{array}\right]=\left[\begin{array}{c} v_{\mathrm{cz},1}\\ \begin{array}{c} v_{\mathrm{cz},2}\\ \vdots \end{array}\\ v_{\mathrm{cz},N} \end{array}\right]\;$$

The absolute velocity of VDI can be obtained by

(12) $$v_{\mathrm{VDI}}=\sqrt{v_{x}^{2}+v_{z}^{2}}\;$$

### Blood Vessel Segmentation

To reduce the data processing time, the *g*_1_(τ) function was used to make a spatial mask for the blood flow region since only the blood flow area is the interested region. This mask can reduce the data processing time by more than five times because the flow region may just take a small portion of the whole image area.

Figure S11A (Supporting Information) illustrates the flowchart for masking, which uses the calculated value of *g*_1_(τ) at time lag 1 (*g*_1_(1)) to make the mask. From the top figure of Figure S11B–D (Supporting Information), it can be seen that the initial value of *g*_1_(τ) at the first time lag within blood vessels (region IV) is significantly higher than that of surrounding tissues (region I, II, and III). This is due to the fact that, after clutter rejection, the slow changing signal in the tissue region is removed and the remaining high‐frequency noise would cause a drop from time lag 0 to time lag 1 and a fast decorrelation as well. Therefore, a threshold of 0.9 is used to the *g*_1_(1) map for the purpose of rejecting non‐vessel regions (second row of Figure S11B, (Supporting Information)). The remaining non‐vessel regions can be further rejected by only keeping the largest connected region (third raw of Figure S11B, Supporting Information). Finally, the segmented vessel region is filled and dilated to recover the pixels falsely eliminated during the previous procedures (fourth and bottom rows of Figure S11B, Supporting Information).

The top figure in Figure S11E (Supporting Information) shows the total velocity maps of carotid artery without masking. It is noticed that the non‐vessel region has higher flow speed than vessel region. This is due to the fast drop of the *g*_1_(τ) function since time lag 1 (red curve in Figure S11C, Supporting Information), which is indeed the autocorrelation function of noise and it is meaningless. Therefore, applying a spatial mask can not only help to make the flow image clearer but more importantly reduce the data processing time.

### Numerical Simulation

Numerical simulation was performed using the Field II ultrasound simulation toolkit.^[^ ^42^, ^43^, ^44^ ^]^ Specifically, a 128‐element transducer probe characterized with a pitch of 0.3 mm and center frequency of 7.2 MHz was used to collect the raw data. For the evaluation of speed measurement, a set of moving point scatters with a preset flow speed along the vessel direction were simulated to obtain the radio frequency (RF) data. The diameter of blood vessel was 6 mm and the particle density was set to be 15 per voxel cell (200*200*200 µm^3^). The preset velocity of moving scatters was subjected to parabolic laminar flow profile, which could be described as:

(13) $$V\;\left(r\right)=V_{\max}\;\cdot \left[1-{\left(\frac{r}{R}\right)}^{2}\right]$$

where *V* is the preset velocity varying along the radial position *r*; *V*_max_ is the maximum velocity; *R* denotes the radius of blood vessel.

To verify the capability of 3C‐vUS to reconstruct the 3D‐velocity‐components, four types of blood vessel was mainly simulated, as summarized in Table S1 (Supporting Information). I) To validate the angle‐independency of 3C‐vUS, a tilted blood vessel with a maximum flow speed of 100 mms^−1^ and a tilted angle of β = 20° was simulated at different probe‐to‐vessel angles α (Figure 3A). II) To validate the estimation accuracy of 3C‐vUS, the blood vessel was imaged at different preset flow speeds and a fixed probe‐to‐vessel angle. III) To verify the capability of 3C‐vUS to measure pulsatile flow, the blood vessel was imaged with a time‐varying preset flow speed and a fixed probe‐to‐vessel angle. IV) To test influence of noise on estimation accuracy of 3C‐vUS, various levels of white noise (SNR: 10, 7.5, 5, 2.5, and 0 dB) were added to the reconstructed IQ data prior to velocity analysis. During data acquisition, planewave ultrasonic pulses consisted of 4 excitation cycles were emitted at emission angles of (−10°, 0°, 10°) with a pulse repetition frequency (PRF) of 30 kHz. The in‐phase and quadrature (IQ) data were reconstructed with different numerical apertures and IQ data were then applied to obtain the 3D‐velocity‐components with 3C‐vUS.

### Phantom Experiment

To test the influence of beamforming aperture on the 3D PSF, silica‐based crystal balls (diameter: 50 µm, JIQI, Hongmei Glass Art, Quanzhou, China) embedded in an agarose phantom were scanned along the elevational direction with an interval of 50 µm. Then, these ultrasonic signals were beamformed into B‐mode images with different receiving apertures and applied to reconstruct the 3D PSFs to test how *σ_x_*, *σ_y_* and *σ_z_* change when modifying the in‐plane beamforming aperture.

To evaluate the speed measurement accuracy of blood vessel with different probe‐to‐vessel angles, a circular‐shaped microtube with 5 mm inner‐diameter was buried in agarose phantom and pumped with 1% SiO_2_ solution by a commercial pulsating pump (PLAB2001D, Preclinic, Shanghai, China). The pulsatile frequency of blood flow was set to be 60 beats min^−1^ to match the cardiac cycle in humans. During imaging, the ultrasound probe performed 1s data acquisition at each elevational position (Y), and then was moved ≈0.5 mm along the Y direction to cover a 40 × 40 mm^2^ square region (Figure 4A). During the phantom experiment, planewave ultrasonic pulses consisted of 4 excitation cycles were emitted with emission angles of (−10°, 0°, 10°) so as to perform VDI analysis. The raw data were IQ‐demodulated and then analyzed to reconstruct the 3D‐velocity‐components of the circular flow.

### Data Acquisition of Human Carotid Artery

The carotid artery imaging of a health subject was performed to validate the capacity of 3C‐vUS for in vivo blood flow speed measurement. For the first experiment session, the subject was guided to sit on a chair and an experienced operator acquired the ultrasound data from the subject at different probe‐to‐vessel angles. For the other experiment session, five untrained students were instructed to acquire data from the carotid artery at arbitrary probe‐to‐vessel angles. Note that the raw data was collected at an emission angle of 0° in carotid artery imaging. IQ‐demodulation, beamforming and 3C‐vUS data processing were then carried out to obtain the 3D‐velocity‐components.

The human experiments were approved by the Medical Ethics Committee of Southern University of Science and Technology (Decision number: 20230064), and the written informed consent was obtained from the subject before the carotid artery imaging. The patient safety measurements were performed under the guidelines of the US Food and Drug Administration.

## Conflict of Interest

The authors declare no conflict of interest.

## Author Contributions

Y.W. and J.T. conceived of the technology and designed this study. Y.W. and J.T. developed the theoretical model and analyzed the results. Y.W. and J.T. developed the data processing method, performed numerical simulation, constructed the experimental setup, carried out experiments, and wrote the manuscript. Y.H. and W.C. carried out phantom experiments and analyzed the results. J.T. supervised this study. All authors discussed the results and contributed to the final version of the manuscript.

## Supporting information

Supporting Information

Supplemental Movie 1

Supplemental Movie 2

Supplemental Movie 3

Supplemental Movie 4

Supplemental Movie 5

Supplemental Movie 6

## Data Availability

The data that support the findings of this study are available from the corresponding author upon reasonable request.
