# Supplementary material for: Ultrasound Speckle Decorrelation Analysis‐Based Velocimetry for 3D‐Velocity‐Components Measurement Using a 1D Transducer Array
Source: Adv Sci (Weinh). 2024 Jun 20;11(32):2401173. doi: 10.1002/advs.202401173 (PMC11348193; doi:10.1002/advs.202401173)

Supporting Information

Ultrasound Speckle Decorrelation Analysis-Based Velocimetry for 3D-Velocity-Components Measurement Using a 1D Transducer Array

Yongchao Wang, Yetao He, Wenkai Chen, Jiyong Tan, Jianbo Tang*

**Derivation of the normalized field autocorrelation function for a 3D ultrasound voxel**

The time-varying ultrasound signal of a 3D measurement voxel is the integration of echoes reflected from all moving point scatters within the voxel. Theoretically, the complex ultrasound quadrature signal at time t for a given voxel can be described as:

$sIQ\left( x_{0},y_{0},z_{0},t \right)=R\sum_{i_{s}}^{N_{s}} e^{-\frac{{(x_{i_{s}}\left( t \right)-x_{0})}^{2}}{2\sigma_{x}^{2}}-\frac{{(y_{i_{s}}\left( t \right)-y_{0})}^{2}}{2\sigma_{y}^{2}}-\frac{{(z_{i_{s}}\left( t \right)-z_{0})}^{2}}{2\sigma_{z}^{2}}\cdot e^{i2k_{0}(z_{i_{s}}\left( t \right)-z_{0})}}$ (1)

where, *sIQ* is the complex ultrasound quadrature signal of the measurement voxel; (*x_0_*, *y_0_*, *z_0_*) is the central coordinate of the measurement voxel; *i_s_* is the index of the *i^th^* scatter; (*x_is_*, *y_is_*, *z_is_*) is the coordinate of the *i^th^* scatter; *R* is the reflector factor. We assume that all scatters have the same reflectivity so that *R* is considered as a constant. *N_s_* is the number of moving scatters within the voxel; *σ_x_*, *σ_y_* and *σ_z_* denote the Gaussian profile width at the 1/e value of the maximum intensity of the PSF in the x, y, and z directions, respectively; *k_0_* is the wavenumber of the central frequency of the transducer. Both the amplitudes and phase of ultrasound field signal appear to fluctuate due to the movement of scatters. The fluctuation of ultrasound field can be further quantified by the normalized first-order field temporal autocorrelation function $g_{1}\left( \tau\right)$.

$g_{1}\left( \tau\right)$*=*$E\left[ \frac{\left\langle{sIQ}^{*}(t)sIQ\left( t+\tau\right) \right\rangle_{t}}{\left\langle{sIQ}^{*}(t)sIQ\left( t \right) \right\rangle_{t}} \right]$ (2)

where, *E[…]* means the average over random initial positions of the particles; *<…>_t_* represents an ensemble temporal average; *τ* is the time lag and *** indicates the complex conjugate.

Assuming that all the scatters are moving with the same velocity within the measurement voxel (*v_x_*, *v_y_*, *v_z_*), the ultrasound signal of the given voxel at time (*t+τ*) can be written as:

$sIQ\left( x_{0},y_{0},z_{0},t+\tau\right)=R\sum_{i_{s}}^{N_{s}} e^{-\frac{{(x_{i_{s}}\left( t \right)+v_{x}\tau-x_{0})}^{2}}{2\sigma_{x}^{2}}-\frac{{(y_{i_{s}}\left( t \right)+v_{y}\tau-y_{0})}^{2}}{2\sigma_{y}^{2}}-\frac{{(z_{i_{s}}\left( t \right)+v_{z}\tau-z_{0})}^{2}}{2\sigma_{z}^{2}}\cdot e^{i2k_{0}(z_{i_{s}}\left( t \right)+v_{z}\tau-z_{0})}}$ (3)

where *v_x_*, *v_y_* and *v_z_* are the velocities along the x, y and z direction, respectively. Putting Equation S1,3 in Equation S2, the first order field autocorrelation function $g_{1}\left( \tau\right)$ can be derived to be:

${g_{1}\left( \tau\right)=e}^{-\frac{\left( v_{x}\tau\right)^{2}}{4\sigma_{x}^{2}}-\frac{\left( v_{y}\tau\right)^{2}}{4\sigma_{y}^{2}}-\frac{\left( v_{z}\tau\right)^{2}}{4\sigma_{z}^{2}}}\cdot e^{i2k_{0}\tau v_{z}}$ (4)

**Supporting figures**


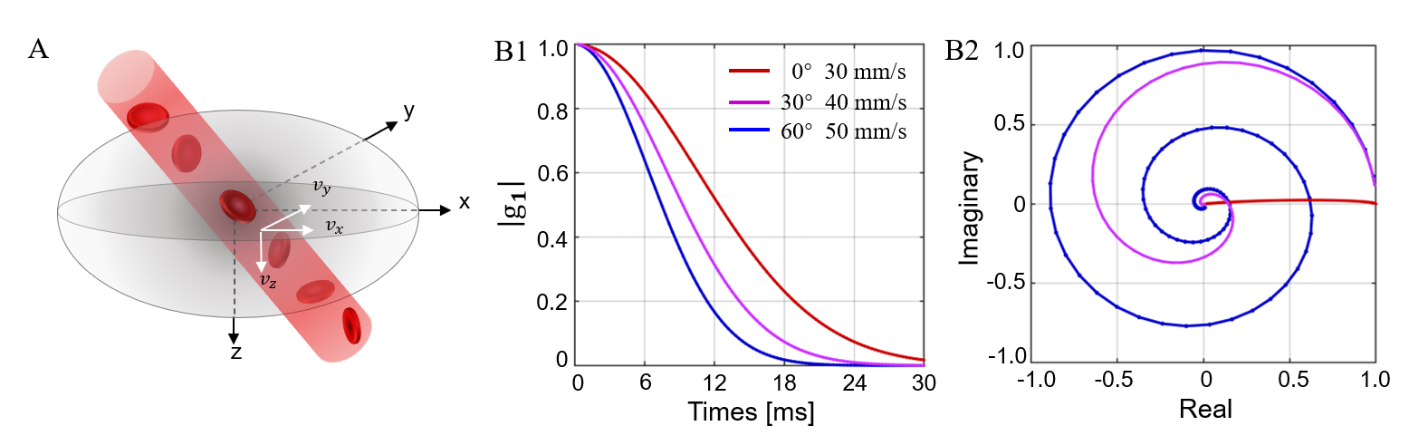


**Figure S1.** Features of $g_{1}(\tau)$ decorrelation. (A) Red blood cells flow through an ultrasound measurement voxel, which is a 3D detection. (B) $g_{1}(\tau)$for different flow angles and speeds in the magnitude plane (B1) and complex plane (B2).


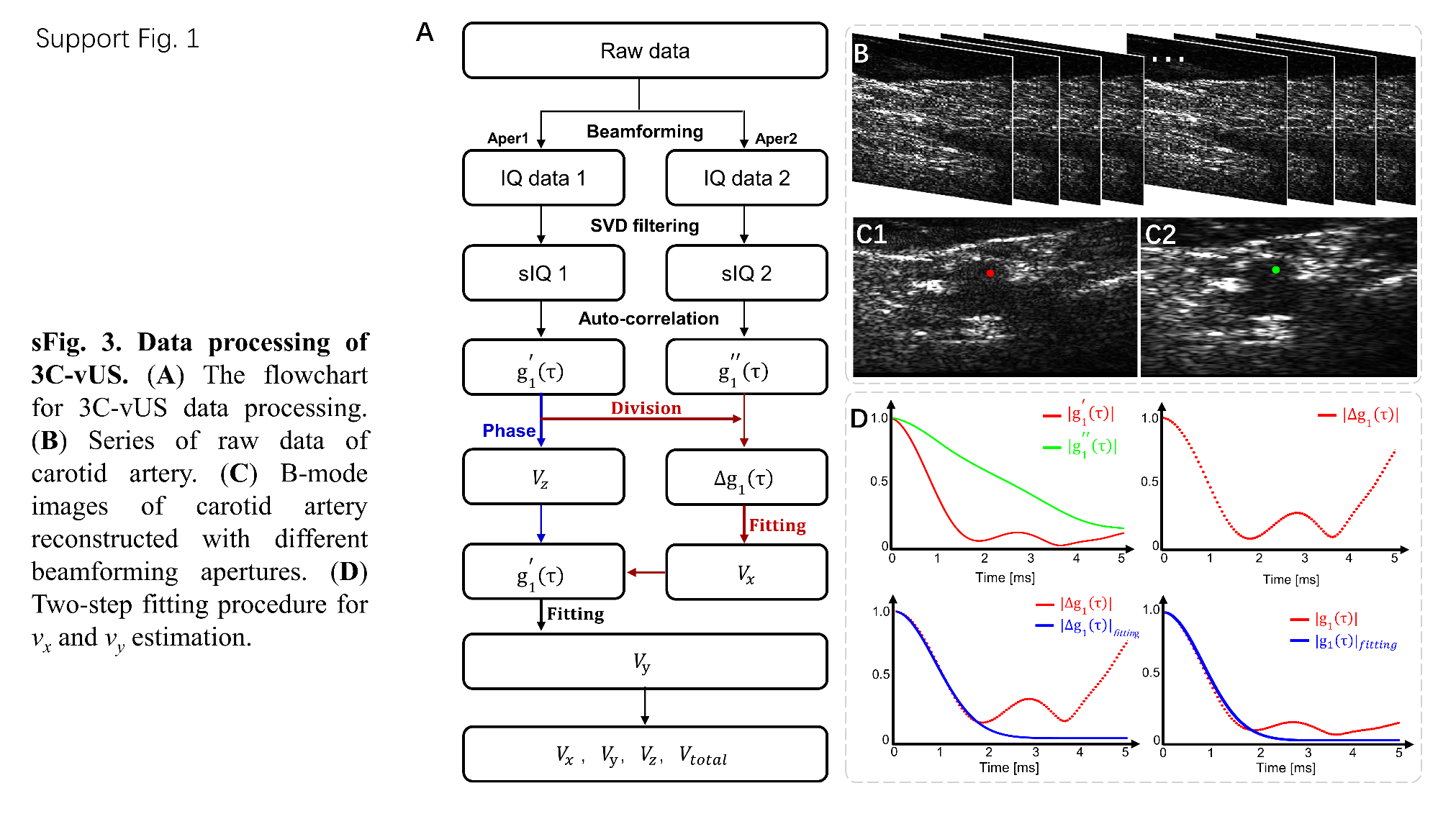


**Figure S2.** The 3C-vUS data processing algorithm. (A) The flowchart for 3C-vUS data processing. (B) A series of ultrasound data were acquired for 3C-vUS data processing. (C) B-mode images of carotid artery reconstructed with different beamforming apertures. (D) Two-step fitting procedures for *v_x_* and *v_y_* estimation.


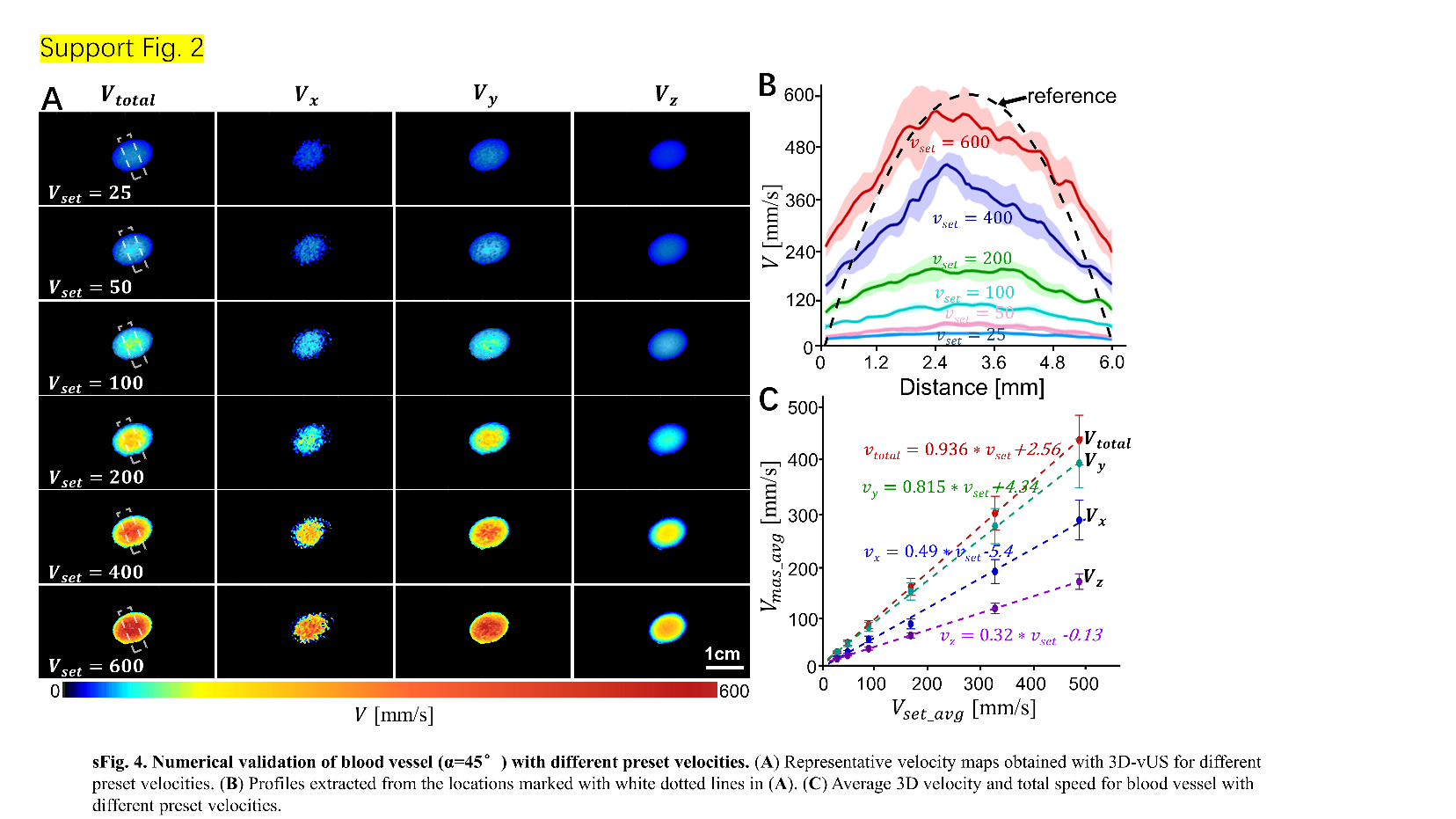


**Figure S3.** Numerical validation for 3C-vUS to measure different preset speeds (α=60°, β=20°). (A) Velocity maps obtained with 3C-vUS at different preset velocities. (B) Blood flow profiles measured at different preset speeds. The black dashed curve shows the preset speed profile for the case of 600 mm/s. (C) Average speeds of the whole cross section for different preset values.


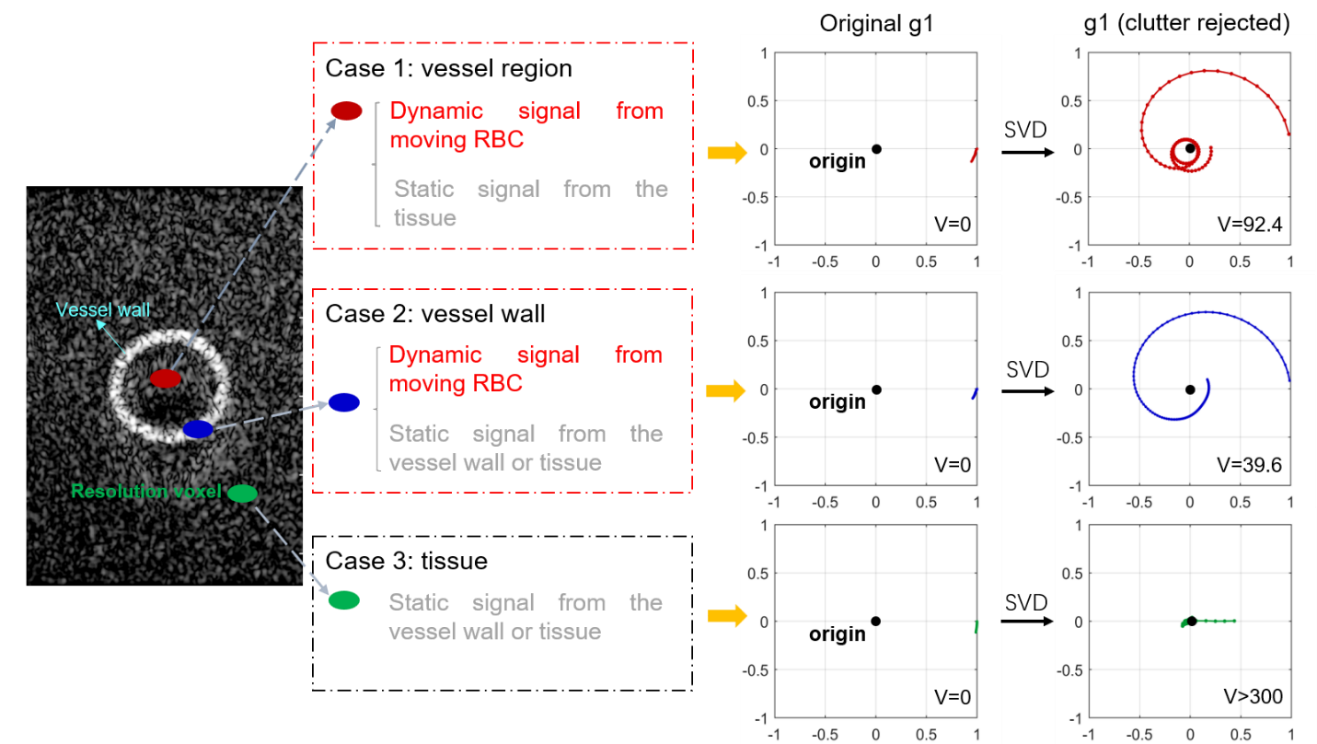


**Figure S4.** The clutter rejection filtering rejects the static signal. The reason the flow speed at the vessel wall (Case 2) is not 0 is due to the limited spatial resolution and clutter rejection filtering. The signal in a measurement voxel near the vessel wall may be composed of static signal from the vessel wall and dynamic signal from the flowing scatterers. The latter component would result in a significant decorrelation of the $g_{1}(\tau)$ function near the vessel wall, hence, non-zero speeds would be reconstructed near the vessel wall region using 3C-vUS.

**
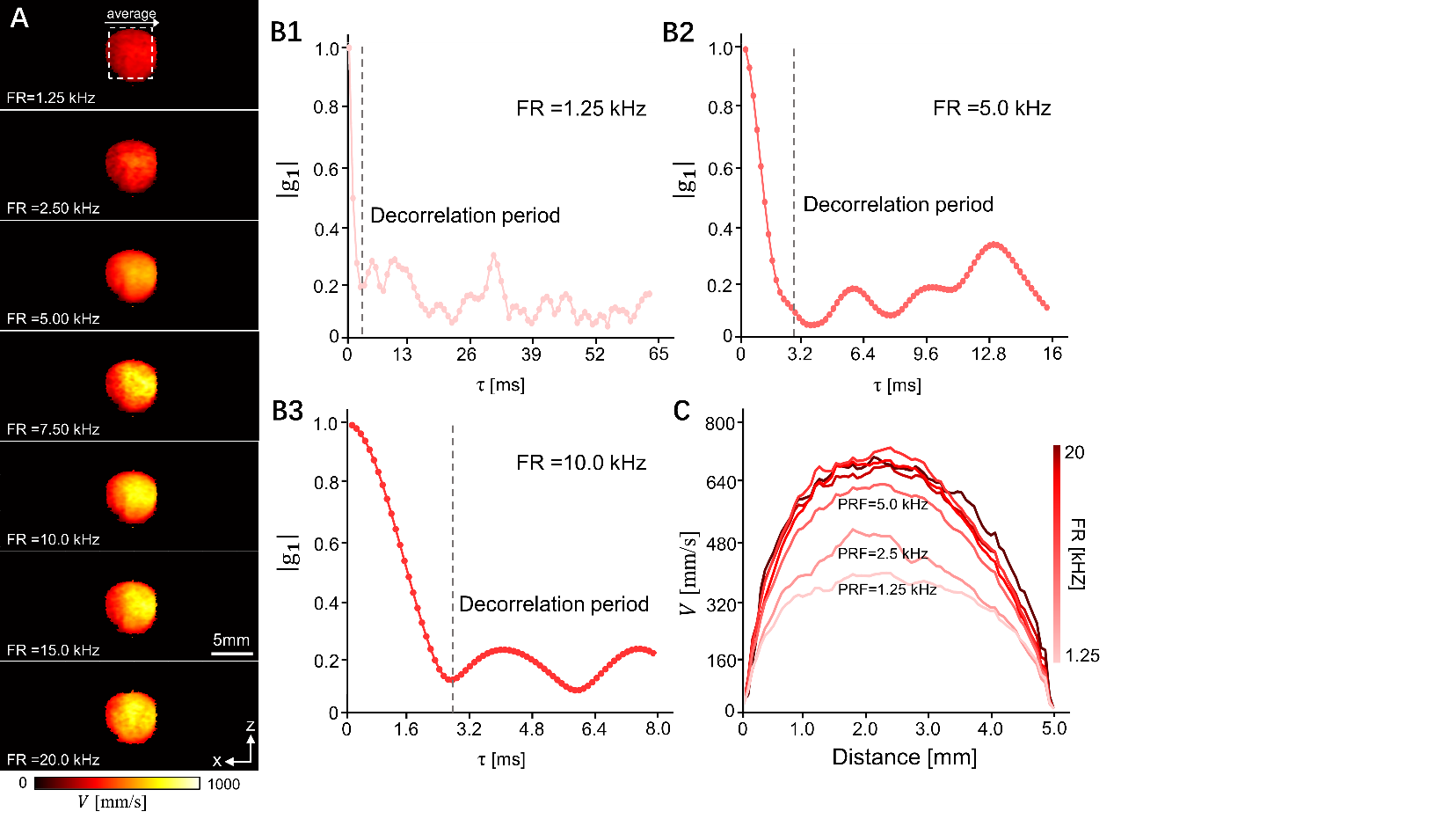
**

**Figure S5.** The influence of frame rate (FR) on velocity estimation. (A) *v_total_* maps for the same blood flow with a FR of 1.25, 2.5, 5.0, 7.5, 10, 15 and 20 kHz, respectively. (B) Representative magnitude decorrelation of $g_{1}(\tau)$ obtained with a FR of 1.25, 5.0 and 10 kHz, respectively. (C) Averaged *v_total_* profiles extracted from the position marked with white dashed box in A. A high FR enables to adequately sample the $g_{1}(\tau)$ within decorrelation period, which helps to improve the estimation accuracy of flow speed.


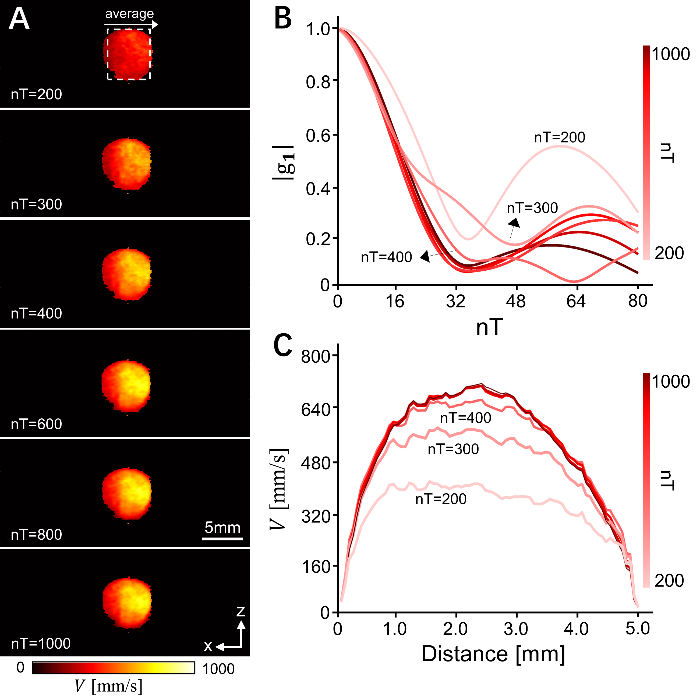


**Figure S6.** The influence of different period window sizes (data acquisition time) on velocity estimation. (A) *v_total_* maps for the same blood flow reconstructed with different period window sizes (nT=200, 300, 400, 600, 800 and 1000 repeats, respectively, Frame rate=15 kHz). (B) The magnitude decorrelation of $g_{1}\left( \tau\right)$ calculated with different period window sizes. (C) Averaged *v_total_* profiles obtained with different period window sizes. We see that when the period window size is shorter than 400 repeats (i.e. 26 ms data acquisition time window at 15 kHz frame rate) would result in a deficient calculation of $g_{1}\left( \tau\right)$ due to the lack of sufficient ensembles, which further leads to incorrect estimation of blood flow speed. Hence, we used a period window size of 600 repeats (i.e. 40 ms acquisition time) for $g_{1}\left( \tau\right)$ in practice.


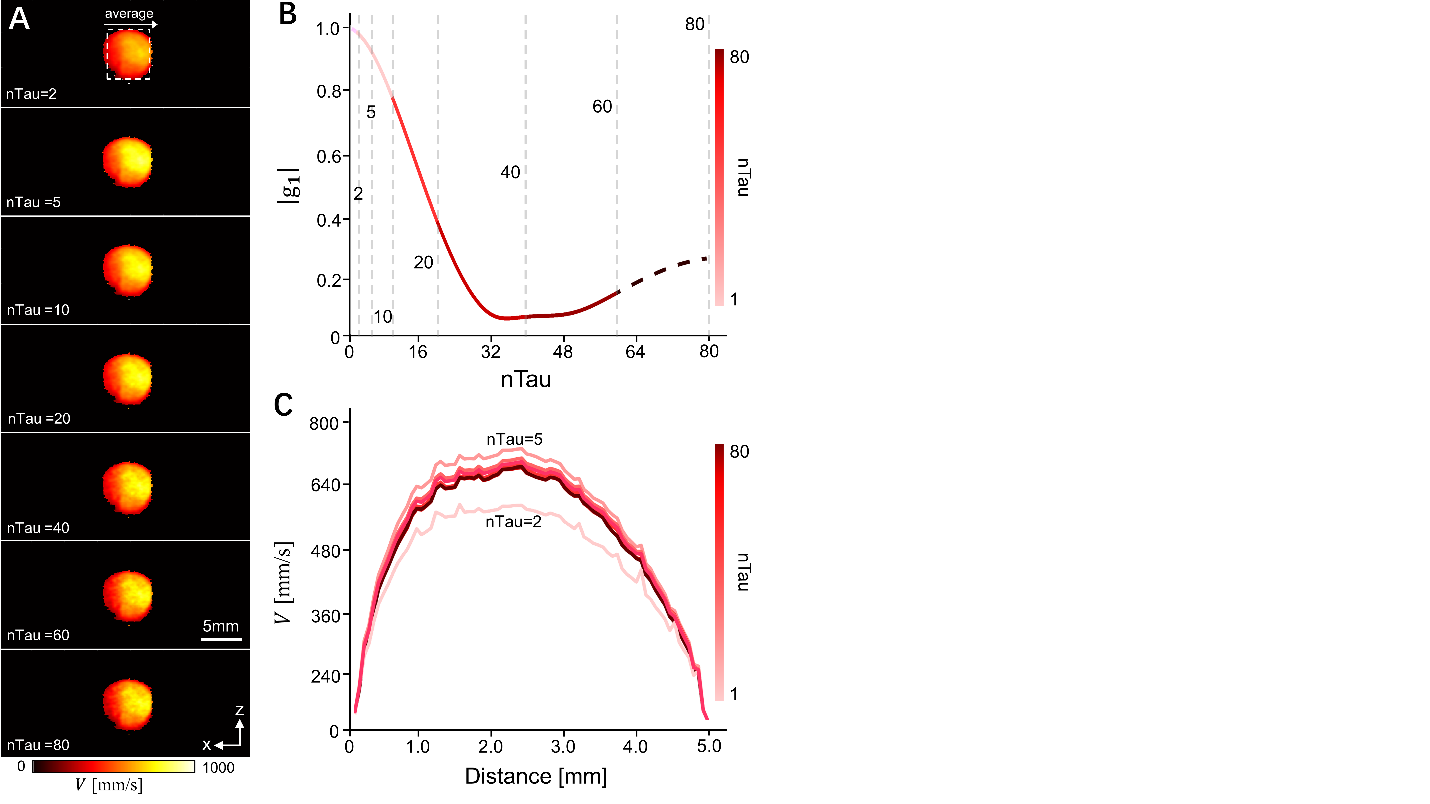


**Figure S7.** The influence of different fitting window sizes on velocity estimation. (A) *v_total_* maps of the same blood flow reconstructed with different fitting window sizes (nTau=2, 5, 10, 20, 40, 60 and 80 repeats, respectively, Frame rate=15 kHz). (B) The magnitude decorrelation of $g_{1}\left( \tau\right)$ calculated for different fitting window sizes. (**C**) Averaged *v_total_* profiles obtained with different fitting window sizes. We see that when the fitting window size is shorter than 10 repeats (i.e. 0.66 ms) would lead to a significant error for *v_total_* estimation, which is due to the lack of information to properly fit the $g_{1}\left( \tau\right)$ decorrelation. As the fitting window size increases, more dynamic information can be used to recover the velocity from the decorrelation function, which leads to more accurate estimation of *v_total_*. It’s also worth mentioning that the fitting window size is not the longer the better, instead, the proper fitting window time shall be just a little longer than the decorrelation time. Hence, in practical implementation considering the carotid artery flow speeds and the system point spread function, we used a fitting window size of 60 repeats (i.e. 4 ms) to recover the velocity information for the $g_{1}\left( \tau\right)$ decorrelation function.


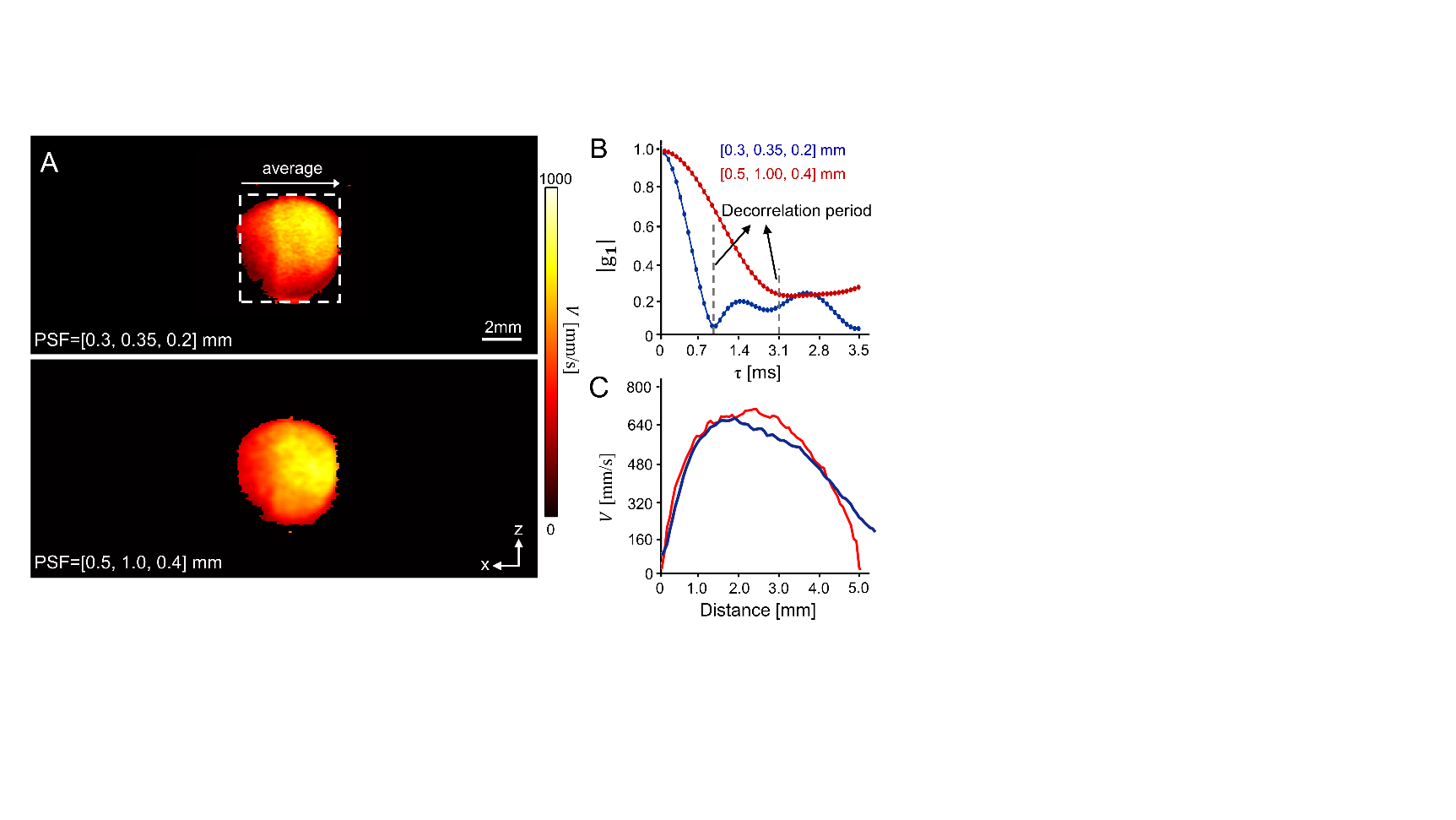


**Figure S8.** The influence of 3D PSF on velocity estimation. (A) *v_total_* maps of the same blood flow reconstructed with different 3D PSFs. (B) The magnitude decorrelation of $g_{1}\left( \tau\right)$ calculated with different 3D PSFs. (C) Averaged *v_total_* profiles obtained with different 3D PSFs. We see that a large 3D PSF will increase the decorrelation period of ultrasound signals, which increases the sampling point of $g_{1}(\tau)$ and helps to improve the estimation accuracy of flow speed.


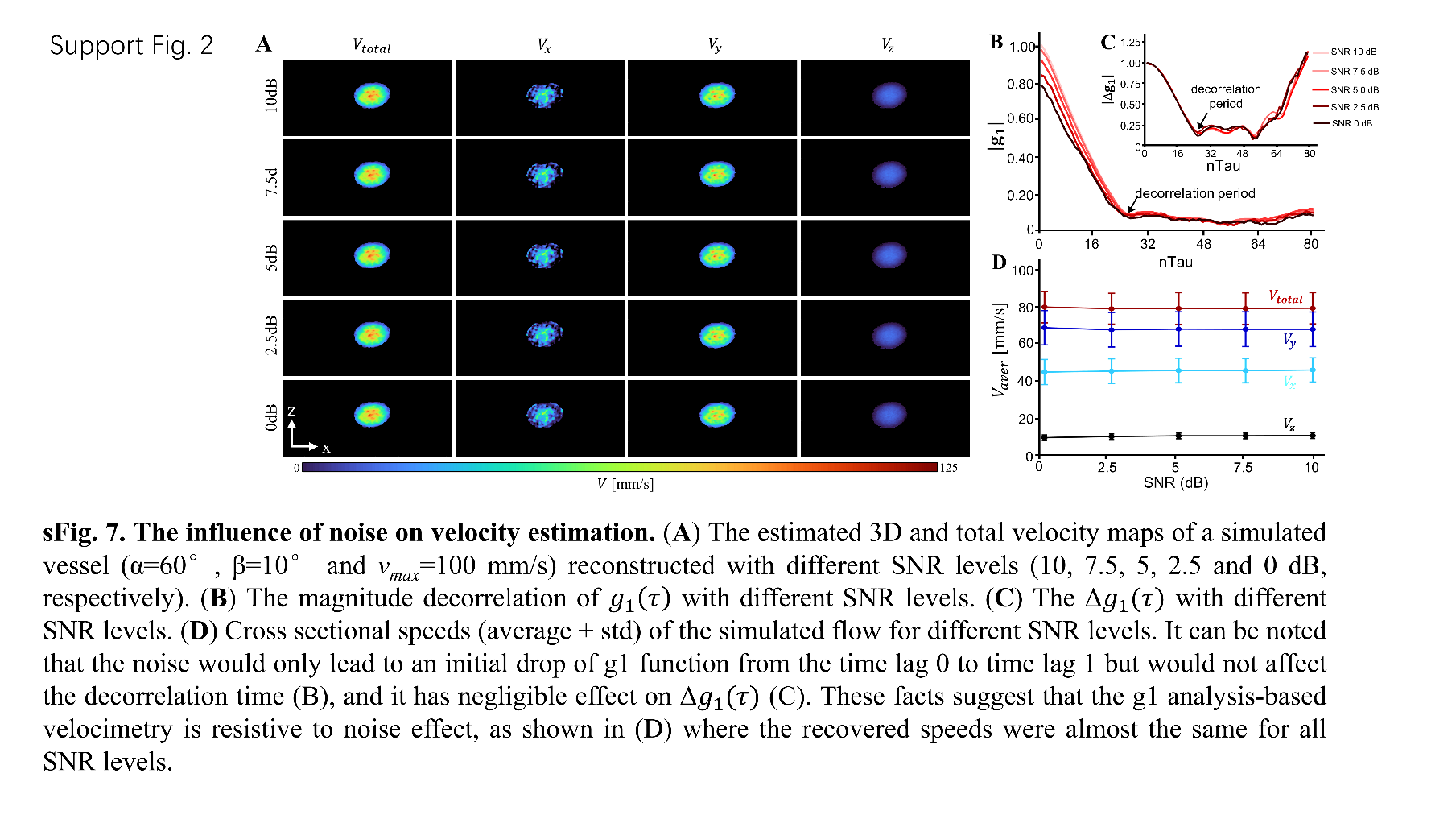


**Figure S9.** The influence of noise on velocity estimation. (A) The estimated 3D and total velocity maps of a simulated vessel (α=60°, β=10° and *v_max_*=100 mm/s) reconstructed with different SNR levels (10, 7.5, 5, 2.5 and 0 dB, respectively). (B) The magnitude decorrelation of $g_{1}\left( \tau\right)$ with different SNR levels. (C) The Δ$g_{1}(\tau)$ with different SNR levels. (D) Cross sectional speeds (average + std) of the simulated flow for different SNR levels. It can be noted that the noise would only lead to an initial drop of $g_{1}\left( \tau\right)$ function from the time lag 0 to time lag 1 but would not affect the decorrelation time (B), and it has negligible effect on Δ$g_{1}(\tau)$ (C). These facts suggest that the $g_{1}\left( \tau\right)$ analysis-based velocimetry is resistive to noise effect, as shown in (D) where the recovered speeds were almost the same for all SNR levels.


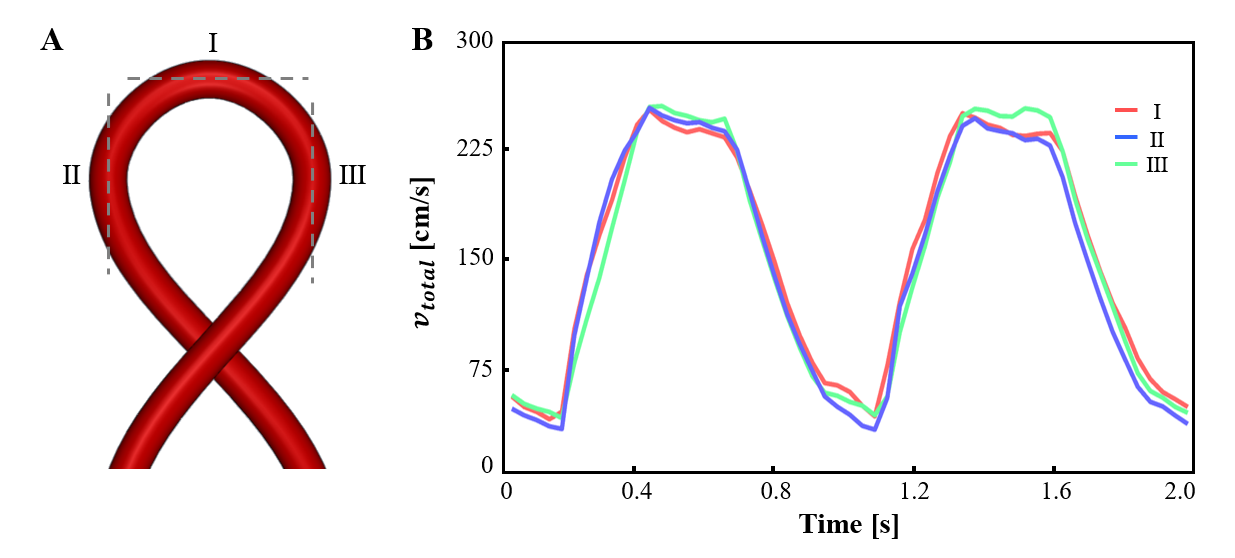


**Figure S10.** Phantom experiment validation with vector Doppler speed measurement. (A) Schematic diagram of the circle-shaped flow for phantom experiment. Vector Doppler imaging (emission angle: -10°, 0°, and 10°) is performed at position Ⅰ, Ⅱ, and Ⅲ marked with gray dashed lines, where the probe-to-vessel angle $\alpha$ is close to 0°. (B) The time-varying total velocity curves obtained with vector Doppler at location Ⅰ, Ⅱ, and Ⅲ. The vector Doppler results measured at these three locations are close to that we obtained with 3C-vUS, as shown in Figure 3E.


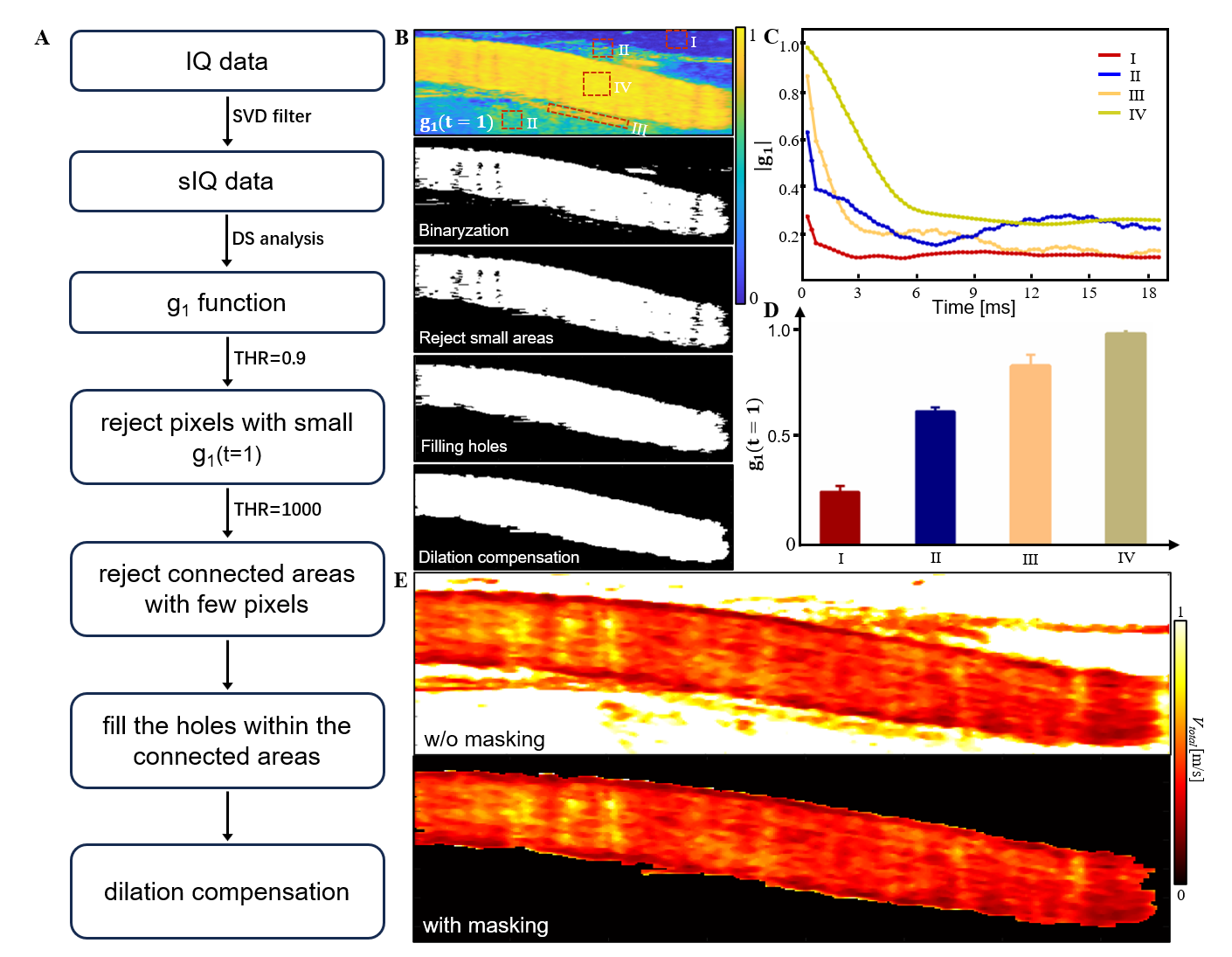


**Figure S11.** The $g_{1}\left( \tau\right)$ based spatial masking for flow measurement. (A) Data-processing flowchart of masking. (B) Step-by-step illustration of the g1-based masking. (C) Magnitude decorrelation of $g_{1}\left( \tau\right)$ obtained from four different regions. Ⅰ, Ⅱ and Ⅲ: non-vessel region; Ⅳ: vessel region. (D) $g_{1}\left( \tau=1 \right)$ at four regions. (E) Flow speed map of carotid artery without (top row) and with (bottom row) masking. Applying a spatial mask can not only help to make the flow image clearer but more importantly reduce the data processing time.


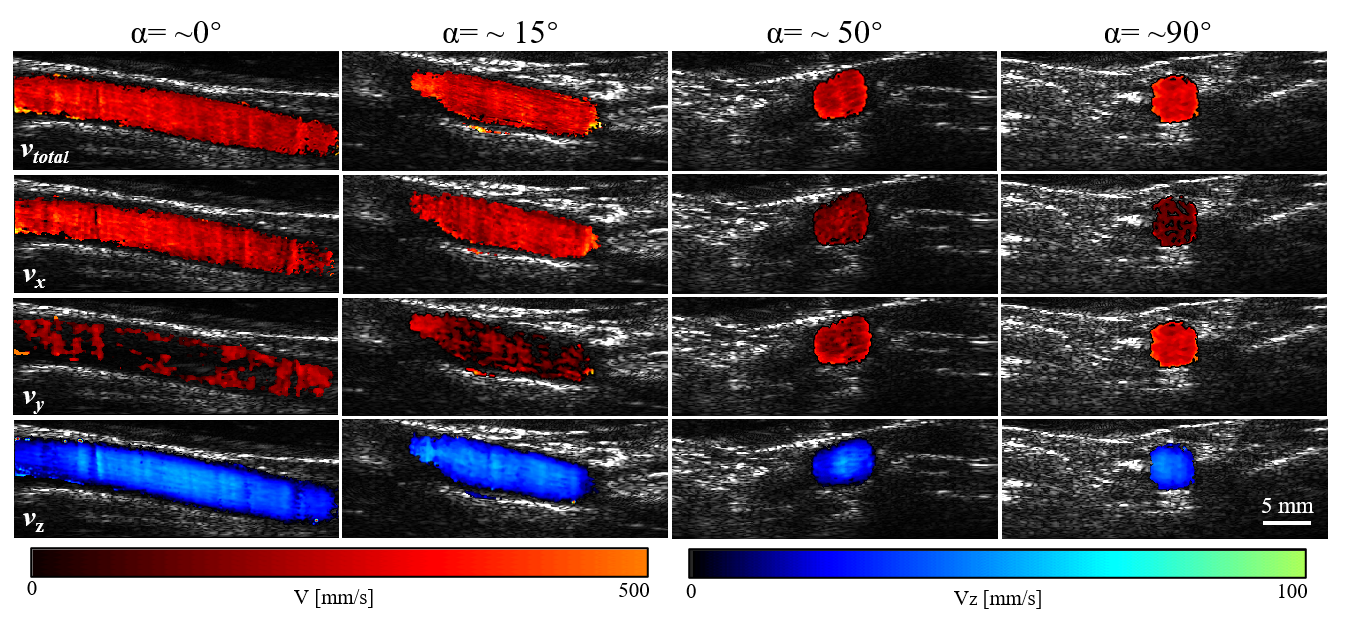


**Figure S12.** *In vivo* imaging of human carotid artery for different angles during the diastolic state. The speed maps of 3D velocity components and the total velocity of the carotid artery during diastolic state at four probe-to-vessel angles.


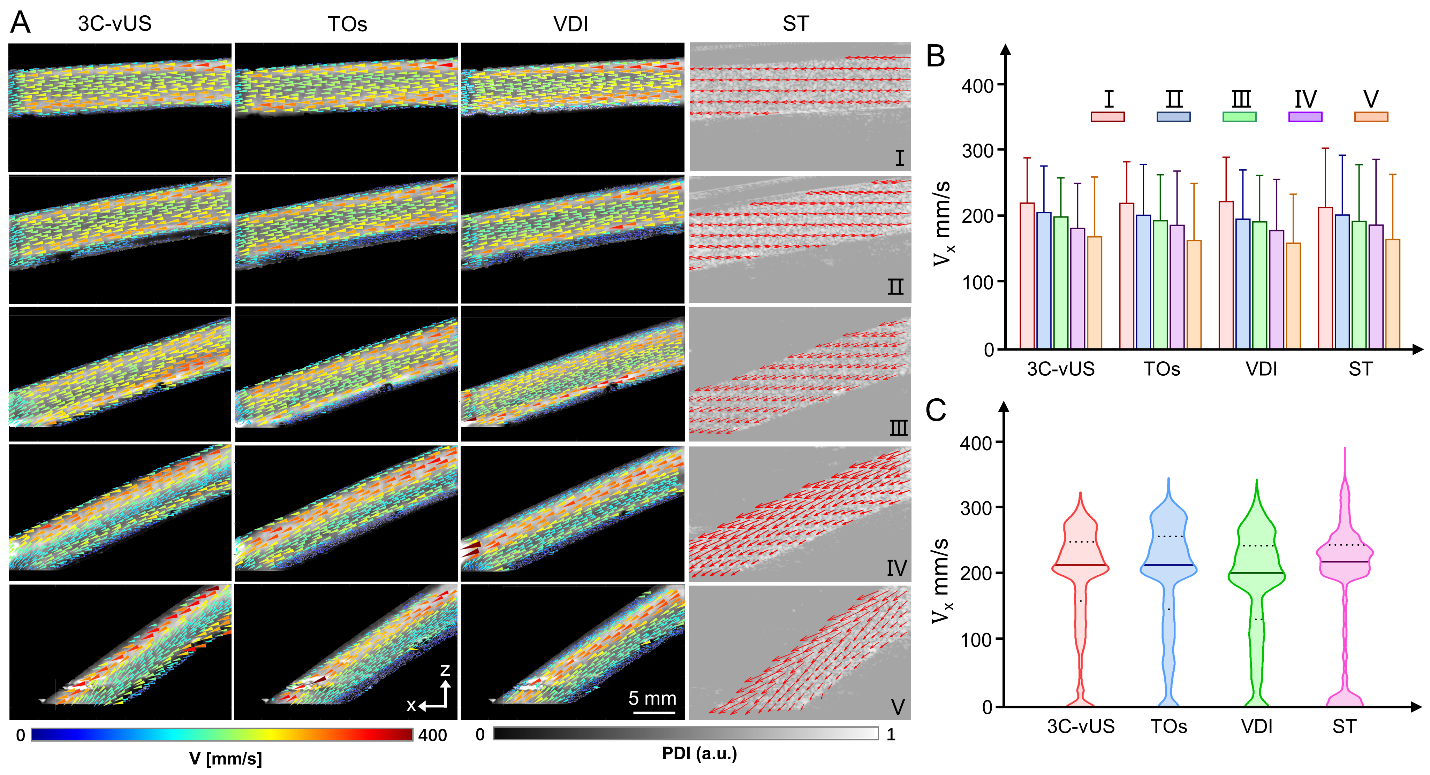


**Figure S13.** Validation of the estimation accuracy of in-plane lateral velocity component *v_x_*. (A) In-plane velocity vector fields obtained with 3C-vUS, transverse oscillation (TOs), vector Doppler imaging (VDI) and speckle tracking (ST) at five beam-to-flow angles. (B) The quantitative analysis (Mean and SD) of the *v_x_* results obtained with 3C-vUS, TOs, VDI and ST at different beam-to-flow angles. (**C**) Violin plots comparing the in-plane velocity level of group Ⅱ cross four techniques. It is apparent that the in-plane velocity vector fields obtained with 3C-vUS is comparable to that of TOs, VDI and ST for all cases. As shown in (B), the quantitative analysis of *v_x_* suggests that all the four techniques exhibit a nearly identical average *v_x_* with a deviation of 6.49% cross five cases, further underscoring the accuracy of 3C-vUS for *v_x_* measurement.


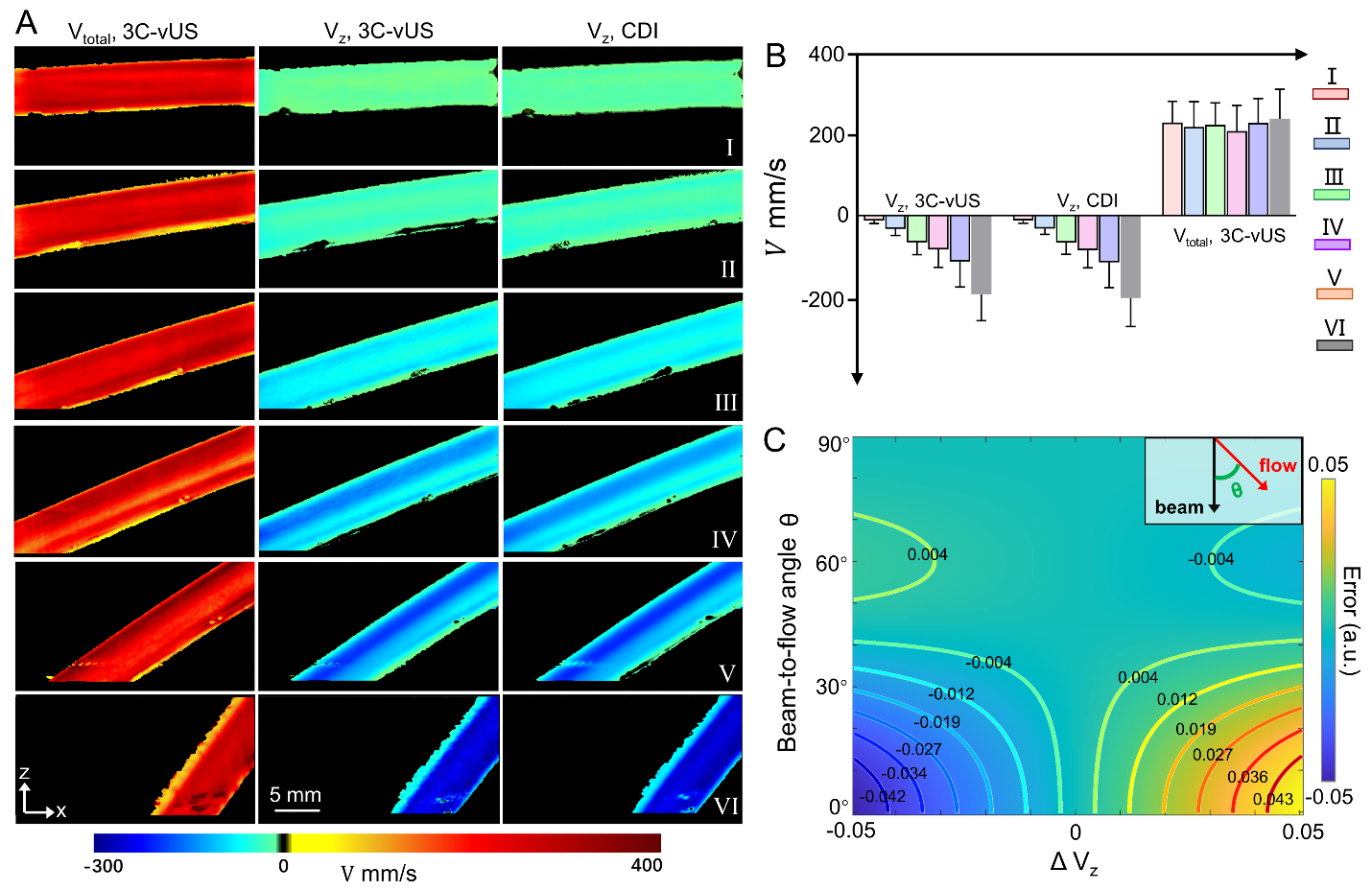


**Figure S14.** The influence of different beam-to-flow angles on the estimation accuracy of *v_total_* and *v_z_*. (A) *v_total_* and *v_z_* maps obtained with 3C-vUS and CDI at six beam-to-flow angles. (B) The quantitative analysis of the *v_total_* and *v_z_* (Mean and SD) obtained with 3C-vUS and CDI at different beam-to-flow angles. (C) Theoretical estimation bias in total flow speed as a function of the deviation in *v_z_* (±5%) and the beam-to-flow angle θ (See the insert of Figure S14C). Note that color Doppler imaging (CDI) is introduced here as a reference standard for 3C-vUS validation. The apparent findings from both the *v_z_* maps and quantitative results suggest that 3C-vUS can achieve comparable *v_z_* measurements to CDI across all cases, with a deviation less than 5%, which suggests the accuracy of 3C-vUS in terms of *v_z_* measurement. One may also note that the averaged *v_total_* remains at 225±16 mm/s for all beam-to-flow angles, further demonstrating that the 3C-vUS technique enables accurate *v_total_* measurement independent of the beam-to-flow angle (θ). It’s worth mentioning that like any imaging technique, 3C-vUS has potential estimation bias compared to the ground truth, which can be derived from the estimation bias of *v_z_*. As shown in (C), significant estimation bias (~5%) in total flow speed occurs primarily when the beam-to-flow angle is less than 10°, with gradual improvement as the angle approaches 90°.


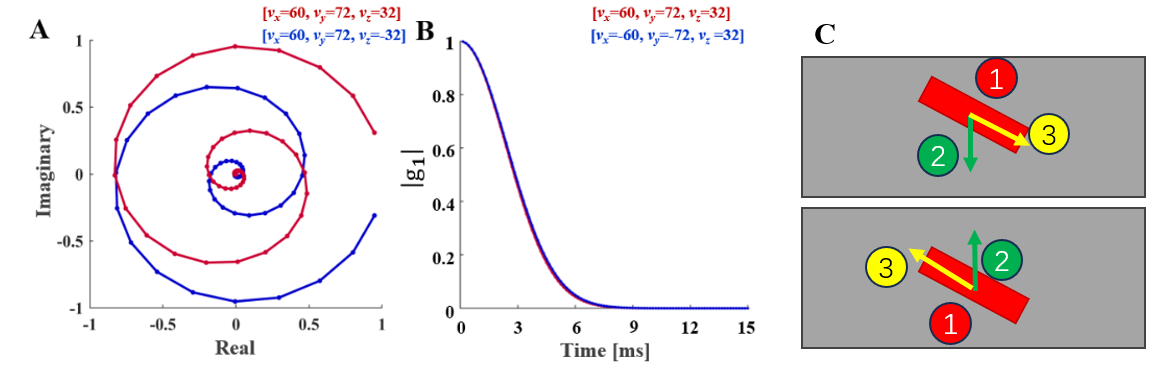


**Figure S15.**  (A) g1 decorrelation curves in the complex plane for two tilted flows with opposite axial flow directions (*v_z_*), which suggests that opposite axial flows have opposite rotating decorrelation paths in the complex plane. (B) g1 decorrelation for two flows with opposite transverse velocity components (*v_x_* and *v_y_*), showing that opposite transverse flows have the same contribution to g1 decorrelation. Thus, in principle, g1 analysis-based method cannot determine the transverse flow direction. (C) A potential solution to obtain the total flow direction using the proposed method is to combine the information from vessel orientation and the axial flow direction.

**Supporting table**


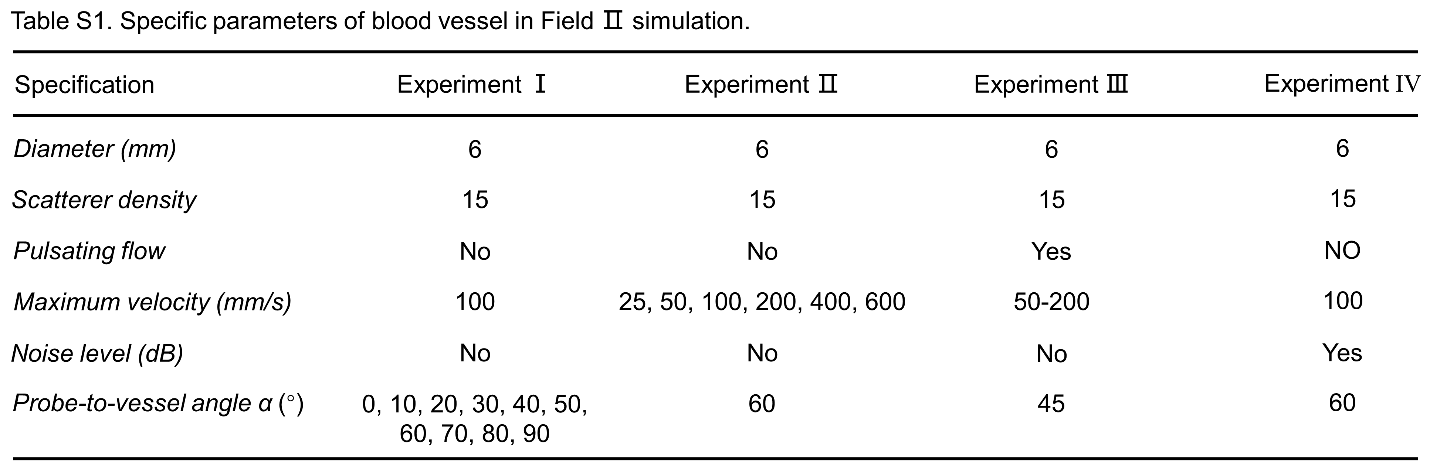

Supplement: Supplementary file 1 — Supporting Information [file ADVS-11-2401173-s005.docx]
